# The polymorphism analysis and epitope predicted of Alphapapillomavirus 9 E6 in Sichuan, China

**DOI:** 10.1186/s12985-021-01728-4

**Published:** 2022-01-20

**Authors:** Jiaoyu He, Qiufu Li, Shiyu Ma, Tianjun Li, Yuning Chen, Yiran Liu, Yanru Cui, Jianying Peng, Yunfan Shi, Xia Wei, Xianping Ding

**Affiliations:** 1 Key Laboratory of Bio-Resources and Eco-Environment of Ministry of Education, College of Life Sciences, Sichuan University, Chengdu, 610065 Sichuan People’s Republic of China; 2 Chongqing Nanchuan Biotechnology Research Institute, Bio-Resource Research and Utilization Joint Key Laboratory of Sichuan and Chongqing, Sichuan and Chongqing, People’s Republic of China; 3 College of Phamaceutical Sciences, Southwest University, Chongqing, 400000 People’s Republic of China; 4 Department of Medical Laboratory, Xindu District People’s Hospital of Chengdu, Chengdu, 610065 Sichuan People’s Republic of China; 5 Institute of Medical Genetics, College of Life Sciences, Sichuan University, Chengdu, 610064 China

**Keywords:** Human Papillomavirus, Alphapapillomavirus 9 genus HPV, *E6* gene polymorphisms, Protein structure, Positive selection site, Antigen epitope

## Abstract

**Background:**

The Alphapapillomavirus 9 (α-9 HPV) is a member of the Alphapapillomavirus genus and Papillomaviridae family. These viruses are almost all carcinogenic HPV, which is closely related to 75% of invasive cervical cancer worldwide, and has a high prevalence in Sichuan. The carcinogenic function is mainly realized by its E6 oncoprotein.

**Methods:**

Cell samples were collected by cervical scraped for HPV detecting and typing. HPV-16, HPV-31, HPV-33, HPV-52, HPV-58 5 α-9 genus HPV subtype positive samples were selected, their *E6* gene was sequenced and analyzed. The positive selection sites of HPV *E6* genes were estimated by PAML 4.8 server. The secondary and tertiary structure of E6 protein were predicted by PSIPred and Swiss-model. The T-cell antigen epitopes of E6 protein were predicted by IEDB.

**Results:**

α-9 HPV has a high prevalence in Sichuan, China. From 2012 to 2017, 18,067 cell cervical samples were collected, and 3135 were detected with α-9 HPV infection. Among which, 250 cases HPV-16 *E6*, 96 cases HPV-31 *E6*, 216 cases HPV-33 *E6*, 288 cases HPV-52 *E6* and 405 cases HPV-58 *E6* were successfully amplified, 17, 6, 6, 13, and 4 non-synonymous nucleotide mutations were respectively detected in HPV-16, 31, 33, 52, and 58 *E6*, 7 positive selection sites of α-9 HPV E6 were selected out (D32E of HPV-16 E6, K35N, K93N and R145I of HPV-33 E6, K93R of HPV-52 E6, K93N and R145K of HPV-58 E6). The structure and antigen epitopes of E6 protein with amino acid substitution differ from those of wild-type E6 protein, especially for the mutation located in the E6 positive selection site.

**Conclusions:**

HPV *E6* nucleotide non-synonymous mutation in the positive selection site influence the protein structure and decrease the antigen epitopes affinity of the E6 protein overall, making it more difficult for the HPV-infected cells to be detected by the immune system, and enhancing the HPV adaptability to the environment. Mutations influence the validity of HPV clinical diagnostic probes, the polymorphism analysis of α-9 HPV E6 enrich the data of HR-risk HPV in Sichuan China, and the detection probes designed with the polymorphism data in mind can improve the efficiency of clinical detection; Mutations influence epitopes affinity, the association of E6 polymorphism and epitope affinity can improve the design of therapeutic vaccine with good immunity and high generality antigen epitope; The above study all provide a good theoretical basis for the prevention and treatment of HPV-related diseases.

**Supplementary Information:**

The online version contains supplementary material available at 10.1186/s12985-021-01728-4.

## Introduction

Cervical cancer is the second most common cancer among women aged 15–44, 99.7% of the cervical cancers were found to be associated with high-risk (HR) Human Papilloma Virus (HPV) persistent infection [1]. The three main genera are α, β, γ, of which α genus is associated with anal and oral mucosal infection. α-9 genus HPV is almost all carcinogenic HPV, causing 75% invasive cervical cancer worldwide, and its carcinogenicity is mainly realized by E6 and E7 early proteins encoded by HPV *E6* and *E7*. The carcinogenicity of HPV E6 protein is more evident than that of E7 protein, in terms of the cell cycle changes and the efficiency of HPV infected cells to permanent biochemical transformations [2, 3]. Without E7, E6 can connect and ubiquitin degradation the p53 protein via E6AP, interfere the cell cycle, activate telomerase and reverse transcriptase to accumulate the mutations, for infected cells immortalization and maintain immortalization, which is closely related to the function of HPV immortalization, cell transformation, and carcinogenesis [4–7].

At present, there is no specific drug for HPV treatment and mainly relies on the body's immune system to detect and eliminate the virus. Human leukocyte antigen (HLA) has the function of recognizing itself target by recognizing and stimulating CD8+ cytotoxic T lymphocytes (CTL), CD4+ helper T lymphocytes (Th) as well as binding antigen polypeptide to regulate the immune response, control and eliminate HPV infection [8–11]. The antigen epitopes are composed of specific amino acid sequences, are the targets of immune rejection [12–14]. HPV E6 protein has been considered as a potential target for the activation of T cells in immune response strategies and maybe an ideal target for HPV therapeutic vaccines [15, 16].

HPV is a high infectious and mutable virus, with different epidemic trends and mutation types in different regions and populations [17]. The polymorphism of HPV *E6* oncogene is strong, its non-synonymous mutation changed E6 protein amino acid composition, which may relate to the differences in immune response and pathogenicity [18]. Some mutant strains can even fix their genes by mutations, enhancing their adaptability to the environment and changing its infection rate [19]. For example, L83V (L90V) of HPV-16 E6 in Swedish and Italian populations as well as D25E (D32E) of HPV-16 E6 in Japanese populations have been proven to be associated with the progression of cervical cancer [20–23]. In the current HPV vaccine design targeting E6 protein, the E6 mutants have almost never been considered. In the epitope-specific vaccine designed by Kelly L, the body's long-active T-cell response to HPV-18 was induced by targeting the reference sequence of HPV E6 and E7. Due to the relatively rapid mutation rate of HPV, the host response capacity, malignant tumor prevention and therapeutic efficacy of vaccine were changed great [15].

HPV has strong regional and population differences, the prevalence of α-9 HPV and the harmfulness of E6 oncoprotein are extremely high, and E6 polymorphism is closely related to the difference of immunogenicity, adaptability, and pathogenicity. Therefore, it’s urgent to study the genetic diversity, positive selection sites, antigen epitope, the protein structure of α-9 HPV E6 for providing data to realize the effective prevention and control of the disease in this region.

## Materials and methods

### Samples resource

The study was ethically approved by the Education and Research Committee and Ethics Committee of Sichuan University, Sichuan, China. Eighteen thousand sixty-seven specimens were randomly collected from January 2012 to December 2017 in Chengdu Women and Children's Center Hospital, Chengdu Jinjiang District Women and Children's Hospital, Angel Women's and Children's Hospital, Affiliated Hospital of Sichuan Reproductive Health Research Center, Sichuan Reproductive Health Research Center Affiliated Hospital, Shuangnan Hospital, Chengdu Song zi niao Sterility Hospital, Infertility Hospital Affiliated to Chengdu Medical College and Chengdu Jinsha hospital. Before sample collection, written informed consent was obtained from all patients or their guardians, and patient privacy is strictly protected. The cell specimens were collected randomly by cervical scraped and placed in − 20 °C antiseptic buffer (9 g NaCl, 10 g C_6_H_5_CO_2_Na, 1 L H_2_O).

### Genomic DNA extraction and HPV typing

HPV DNA was extracted and evaluated using the Human Papillomavirus Genotyping Kit For 23 Types (Yaneng Bio, Shenzhen, China) according to the manufacturer’s guidelines.

### PCR amplification and variant identification

The primers of α-9 HPV *E6* were designed by PRIMER version 5.0 and NCBI (National Center for Biotechnology Information) Primer Blast based on the reference sequences, the primers and reference sequences used for the molecular characterization analysis of α-9 HPV *E6* were shown in Additional file 1: Table S1 and synthesized by TSINGKE (Chengdu, China). The PCR reaction system consists of 5 µl HPV DNA, 13.1 µl ddH_2_O, 1 µl primers, 0.4 µl TransTaq DNA polymerase, 2.5 µl dNTPs, and 3 µl buffer. The reaction conditions were shown in Additional file 1: Table S1. The PCR products were visualized by gel electrophoresis in 2% agarose gel (Sangon Biotech Co., Ltd.). The target products of *E6* were purified and sequenced by TSINGKE at least twice (Chengdu, China).

### Sequence analysis

#### Genetic polymorphisms analysis of α-9 HPV *E6* gene

The successfully amplified sequences was sequenced, and the sequences were analyzed by NCBI BLAST, Premier5, and DNAMAN5.2.2. Nucleotide mutations of α-9 HPV *E6* sequence were determined according to the reference sequence in GenBank (Additional file 1: Table S1). Chi-square test was used to confirm the significance of data differences, and *P* < 0.05 was considered as significant differences between the data.

#### Selective pressure analysis of α-9 HPV *E6*

Phylogenetic Analysis by Maximum Likelihood 4.8 (PAML 4.8, http://abacus.gene.ucl.ac.uk/software/paml.html) was used to calculate the ratio (ω = dN/dS) between non-synonymous mutation rate (dN) and synonymous mutation rate (dS) to determine the α-9 HPV *E6* gene-positive selection sites.

#### Amino acid composition and protein structure analysis of α-9 HPV E6

Mega6.0 software was used to translate the *E6* nucleotide sequence into the E6 protein sequence. PSIPred (http://bioinf.cs.ucl.ac.uk/psipred/) and Swiss-model were used to analyze the secondary and tertiary structure of E6 protein.

#### T-cell antigen epitopes predicted analysis of α-9 HPV E6 protein

According to the Chinese major histocompatibility complex database (dbMHC) average frequency of HLA alleles, 13 HLA-I and 6 HLA-II alleles were selected (Additional file 1: Table S2). Based on the selected HLA alleles, the T-lymphocyte epitopes of α-9 HPV E6 protein were predicted by IEDB resource (http://www.iedb.org/). According to the method recommended by IEDB, lower the percentile rank (PR) of antigen epitopes is better the affinity, peptides with PR < 1.0 for HLA-I and peptides with PR < 5.0 for HLA-II were deemed to meaningful as well as selected for further analysis.

## Results

### The prevalence of α-9 HPV in Sichuan

**Fig. 1:**
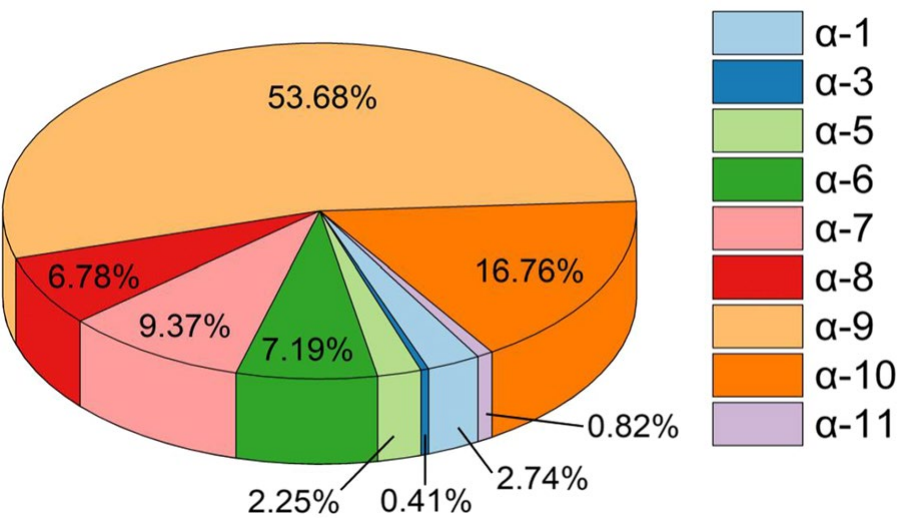
Distribution of different HPVs positive samples from 2012 to 2017

Out of 18,067 samples, 6092 positive results were detected and 4466 were HR HPV, all of which belonged to α genus. The HPV positive samples of α-1, α-3, α-5, α-6, α-7, α-8, α-9, α-10, α-11 are 167 (2.74%), 25 (0.41%), 137 (2.25%), 438 (7.19%), 571 (9.37%,), 413 (6.78%), 3270 (53.68%), 1021 (16.76%), 50 (0.82%) respectively (Fig. 1). α-9 HPV accounting for 73.22% of HR HPV positive samples. Due to the small positive sample sizes of HPV-35 and HPV-67, five other HPV type (HPV-16, HPV-31, HPV-33, HPV-52, HPV-58) were selected for subsequent studies.

### Nucleotide polymorphisms and selective pressure analysis of α-9 HPV E6

250 HPV-16 *E6* were successfully amplified, 162 (64.80%) HPV-16 *E6* samples were variants, and 17 non-synonymous mutations were detected. 96 HPV-31 *E6* were successfully amplified, 68 (70.80%) variants and 6 non-synonymous mutations were detected. 216 HPV-33 *E6* were successfully amplified, 76 (35.19%) variants and 6 non-synonymous mutations were detected. 288 HPV-52 *E6* were successfully amplified, 250 (86.80%) variants and 13 non-synonymous mutations were detected. 405 HPV-58 *E6* were successfully amplified, 356 (87.90%) variants and 4 non-synonymous mutations were detected. Details of α-9 HPV *E6* nucleotide polymorphisms were shown in Tables 1, 2, 3, 4 and 5. All the sequences were submitted to the GenBank, and accession numbers were obtained. (HPV16E6: MZ803036-MZ803058, HPV31E6: MZ803026-MZ803035, HPV33E6: MZ576479-MZ576485, HPV52E6: MZ803059-MZ803078, HPV58E6: MZ803079-MZ803087).

**Table 1:** Nucleotide mutation and amino acid substitution in HPV-16 E6

	HPV-16 E6																				
No	1	2	3	4	5	6	7	8	9	10	11	12	13	14	15	16	17	18	19	20	21
Location	2	12	30	49	94	96	96	96	101	103	103	111	185	192	194	196	253	268	313	360	452
Mutation	T–A	G–A	G–A	A–G	G–A	T–A	T–G	T–C	T–G	T–G	T–C	T–C	G–T	G–T	A–G	C–G	C–T	T–G	G–C	A–C	G–C
Frequency (%)	0.80	3.20	0.80	0.80	0.80	0.80	49.6	0.80	0.80	0.80	0.80	1.60	0.80	0.80	1.60	0.80	0.80	5.60	0.80	5.60	0.80
Substitution	M1K	–	–	R17G	D32N	D32E	D32E	D32E	I34R	L35V	L35V	–	R62I	–	N65S	P66A	H85Y	L90V	D105H	E120D	R151T

Compared with the HPV-16 E6 reference sequence (NC001526), the mutations are marked with the corresponding bases and amino acid, and those without changes are replaced with a dash (–). No. means the number of nucleotide mutations, Location means the sites of nucleotide mutations, Mutation means the style of nucleotide mutations, Frequency (%) means the percentage of nucleotide mutations, Substitution means the amino acid substitution that occurred by nucleotide mutations

**Table 2:** Nucleotide mutation and amino acid substitution in HPV-31 E6

	HPV-31 E6												
No	1	2	3	4	5	6	7	8	9	10	11	12	13
Location	27	69	141	178	190	194	205	219	228	297	321	368	413
Mutation	T–A	C–T	T–C	C–T	A–G	A–G	T–C	A–G	T–C	G–A	A–G	A–G	C–T
Frequency (%)	8.33	27.08	4.17	31.25	4.17	2.08	4.17	16.67	2.08	31.25	27.08	4.17	35.42
Substitution	–	–	–	H60Y	T64A	K65R	F69L	–	–	–	–	K123R	A138V

Compared with the HPV-31 E6 reference sequence (J04353), the mutations are marked with the corresponding bases and amino acid, and those without changes are replaced with a dash (–). No. means the number of nucleotide mutations, Location means the sites of nucleotide mutations, Mutation means the style of nucleotide mutations, Frequency (%) means the percentage of nucleotide mutations, Substitution means the amino acid substitution that occurred by nucleotide mutations

**Table 3:** Nucleotide mutation and amino acid substitution in HPV-33 E6

	HPV-33 E6							
No	1	2	3	4	5	6	7	8
Location	105	165	221	256	279	338	434	441
Mutation	A–C	A–G	G–C	A–C	A–C	A–G	G–T	T–C
Frequency (%)	19.44	7.87	3.24	7.41	19.44	7.41	15.28	11.57
Substitution	K35N	–	S74T	N86H	K93N	Q113R	R145I	–

Compared with the HPV-33 E6 reference sequence (M12732.1), the mutations are marked with the corresponding bases and amino acid, and those without changes are replaced with a dash (–). No. means the number of nucleotide mutations, Location means the sites of nucleotide mutations, Mutation means the style of nucleotide mutations, Frequency (%) means the percentage of nucleotide mutations, Substitution means the amino acid substitution that occurred by nucleotide mutations

**Table 4:** Nucleotide mutation and amino acid substitution in HPV-52 E6

	HPV-52 *E6*																				
No	1	2	3	4	5	6	7	8	9	10	11	12	13	14	15	16	17	18	19	20	21
Location	61	82	93	118	136	136	230	237	249	255	265	270	277	277	278	315	315	366	381	412	429
Mutation	G–A	C–T	G–C	A–C	C–G	C–T	G–A	T–C	G–T	G–A	G–A	G–A	A–C	A–G	A–G	T–G	T–A	C–A	T–A	G–A	A–G
Frequency (%)	0.70	0.70	0.70	0.70	0.70	2.10	0.70	0.70	85.40	9.70	0.70	0.70	1.40	2.10	84.00	0.70	0.70	0.70	0.70	0.70	2.80
Substitution	E21K	–	–	–	L46V	L46V	R77K	–	–	–	E89K	–	K93R	K93R	K93R	I105M	I105M	N122K	N127I	E138K	–

Compared with the HPV-52 E6 reference sequence (NC001592), the mutations are marked with the corresponding bases and amino acid, and those without changes are replaced with a dash (–). No. means the number of nucleotide mutations, Location means the sites of nucleotide mutations, Mutation means the style of nucleotide mutations, Frequency (%) means the percentage of nucleotide mutations, Substitution means the amino acid substitution that occurred by nucleotide mutations

**Table 5:** Nucleotide mutation and amino acid substitution in HPV-58 E6

	HPV-58 *E6*							
No	1	2	3	4	5	6	7	8
Location	78	94	150	198	258	279	286	434
Mutation	C–T	G–C	A–G	C–T	C–A	A–C	T–C	G–A
Frequency (%)	1.73	0.25	0.49	58.52	0.25	27.41	0.25	3.95
Substitution	–	E32Q	–	–	D86E	K93N	–	R145K

Compared with the HPV-58 E6 reference sequence (D90400), the mutations are marked with the corresponding bases and amino acid, and those without changes are replaced with a dash (–). No. means the number of nucleotide mutations, Location means the sites of nucleotide mutations, Mutation means the style of nucleotide mutations, Frequency (%) means the percentage of nucleotide mutations, Substitution means the amino acid substitution that occurred by nucleotide mutations

Calculated by Codeml software using Naive NEB and Bayes Empirical Bayes models, seven positive selection sites of α-9 HPV E6 were detected, there were D32E of HPV-16 E6, K35N, K93N, R145I of HPV-33 E6, K93R of HPV-52 E6, K93N, R145K of HPV-58 E6. In contrast, no reliable HPV-31 E6 positive selection site was selected out (Table 6).

**Table 6:** Positive section site of α-9 HPV E6

Model	HPV-16	HPV-31	HPV-33	HPV-52	HPV-58
M7	NA	NA	NA	NA	NA
M8	32D**	NA	35 K**, 93 K**, 145R**	93 K**	93 K**, 145R**

M7 means NEB (Naive Empirical Bayes) model, M8 means BEB (Bayes Empirical Bayes) model. When the posterior probability was ≥ 0.9, the BEB method was used to identify the positive selection sites. *P* < 0.05 indicates that the results of M8 model are reliable, two asterisks means a posteriori probability ≥ 0.99, and NA means not apply

### The protein structure analysis of α-9 HPV E6

Nucleotides non-synonymous mutation changed the amino acid composition of protein, which affects the structure of the protein, while the protein function is mainly realized by its structures. With the help of Mega6.0, PSIPred and Swiss-model, the primary, secondary, and tertiary structure difference of α-9 HPV E6 protein reference and mutation sequence were revealed.

**Fig. 2:**
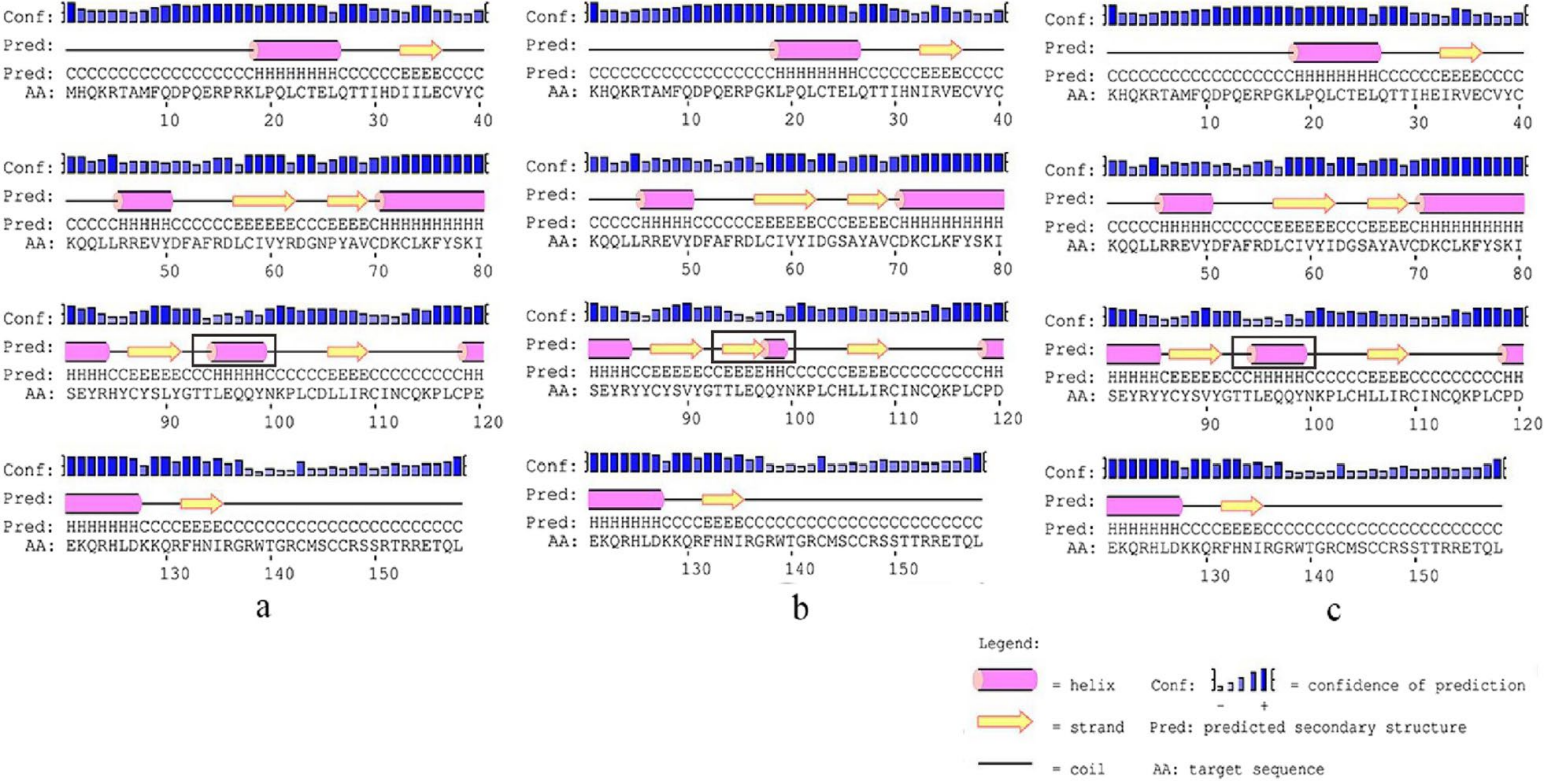
Secondary structure of HPV-16 E6 comparing reference to the variant sequence. *Note* **a** is the secondary structure pattern diagram constructed based on HPV-16 E6 reference sequence; **b** is the secondary structure pattern diagram constructed based on HPV-16 E6-1 mutation sequence; **c** is the secondary structure pattern diagram constructed based on HPV-16 E6-2 mutation sequence, b and c are different in 32th amino acid. The black boxes are the difference areas between the reference and mutation sequence secondary structure.

**Fig. 3:**
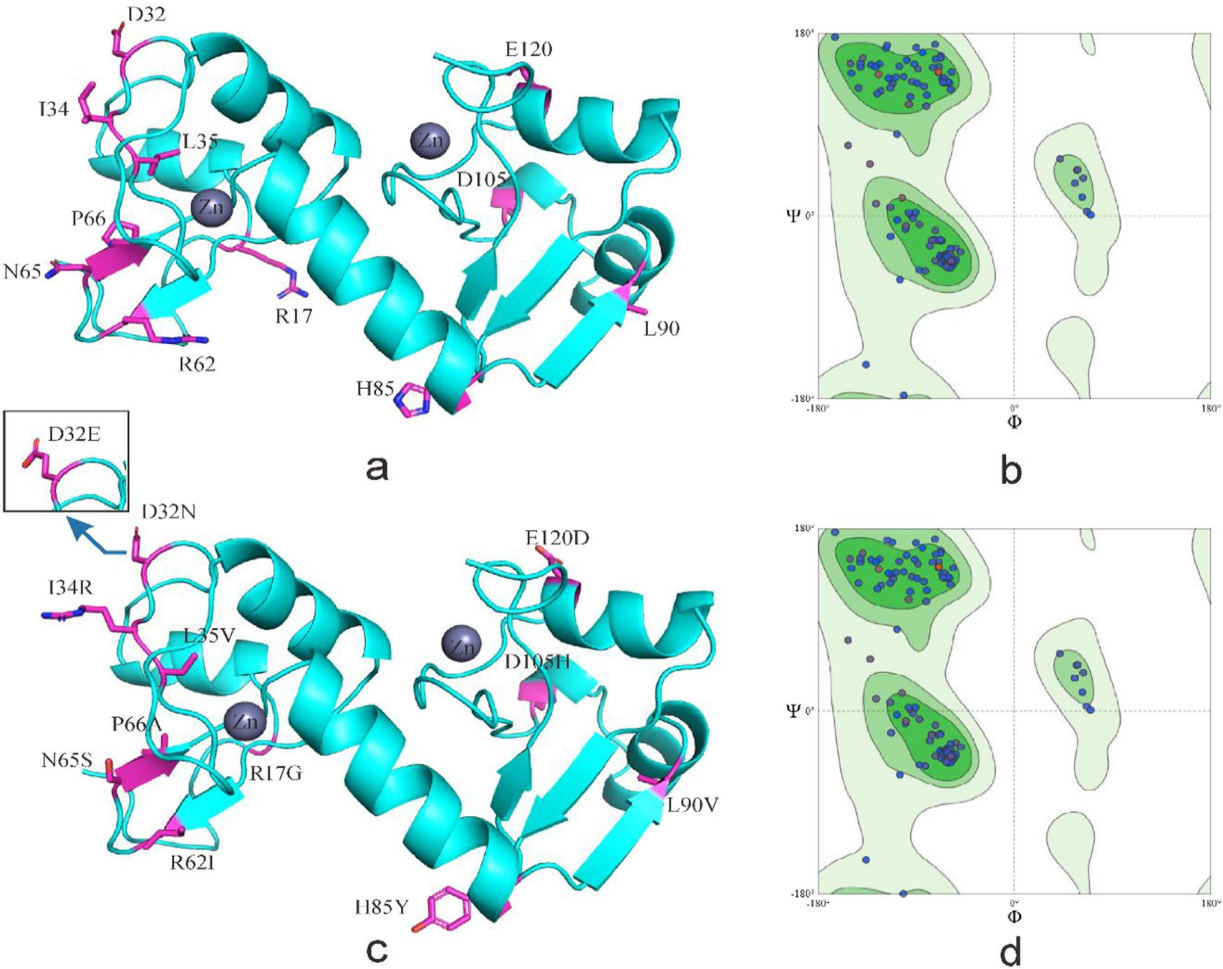
Tertiary structure of HPV-16 E6 comparing reference to the variant sequence. *Note* **a** is the homology modeling structure of HPV-16 E6 reference sequence; **b** is the larlarian diagram of HPV-16 E6 reference sequence homology modeling; **c** is homology modeling structure of HPV-16 E6 mutation sequence; d is the larchian diagram of HPV-16 E6 mutation sequence homology modeling

In HPV-16 E6, I34R, L35V, R62I, P66A and L90V all located in β-fold, E120D, D32N, D32E located in the periphery of the spatial protein structure and close to the active region of znic granules. The amino acid number in the α-helix and β-sheet regions are different in protein reference and mutation sequence. Details are shown in Figs. 2 and 3.

**Fig. 4:**
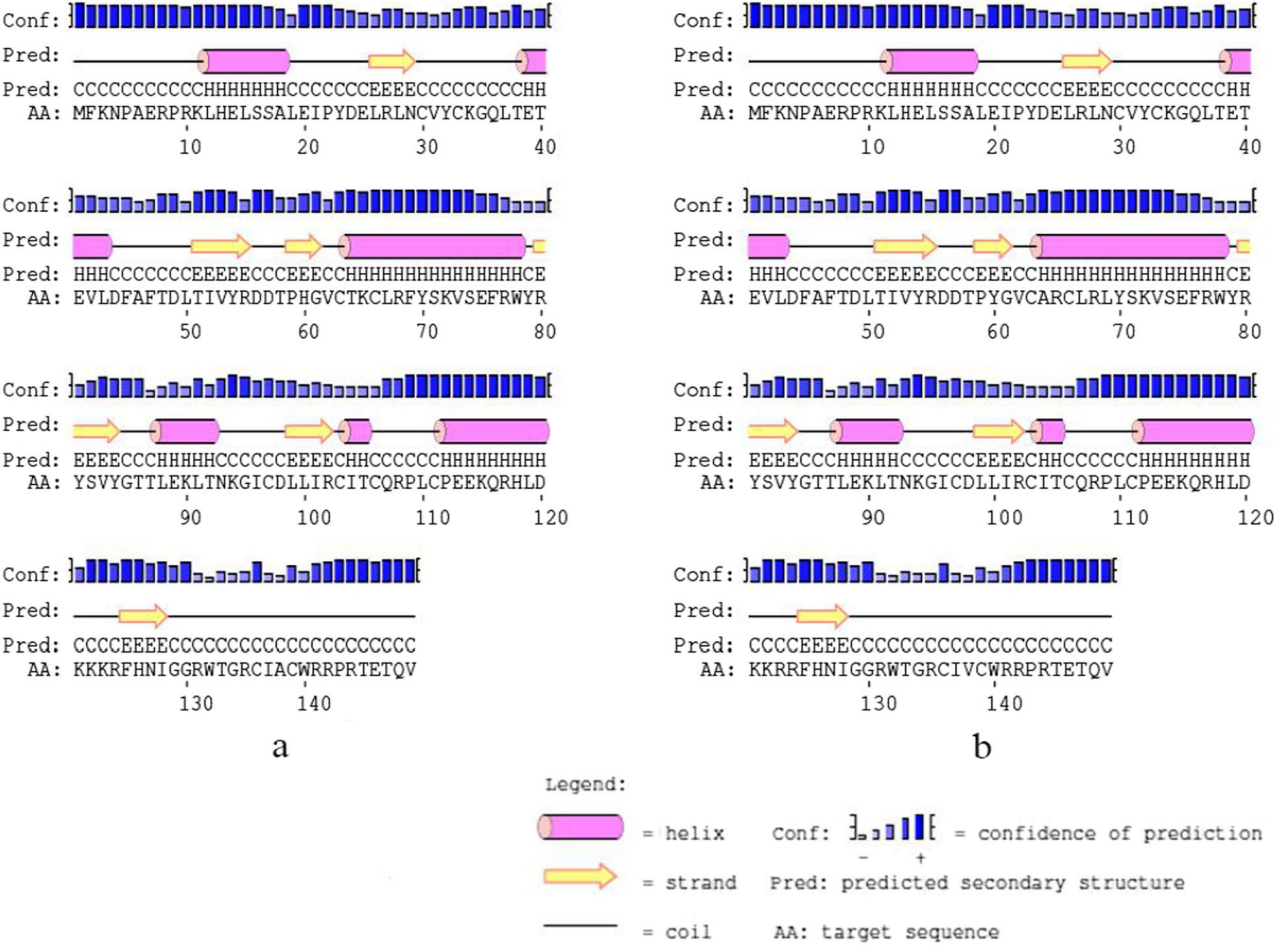
Secondary structure of HPV-31 E6 comparing reference to the variant sequence. *Note* **a** is the secondary structure pattern diagram constructed based on HPV-31 E6 reference sequence; **b** is the secondary structure pattern diagram constructed based on HPV-31 E6 mutation sequence

**Fig. 5:**
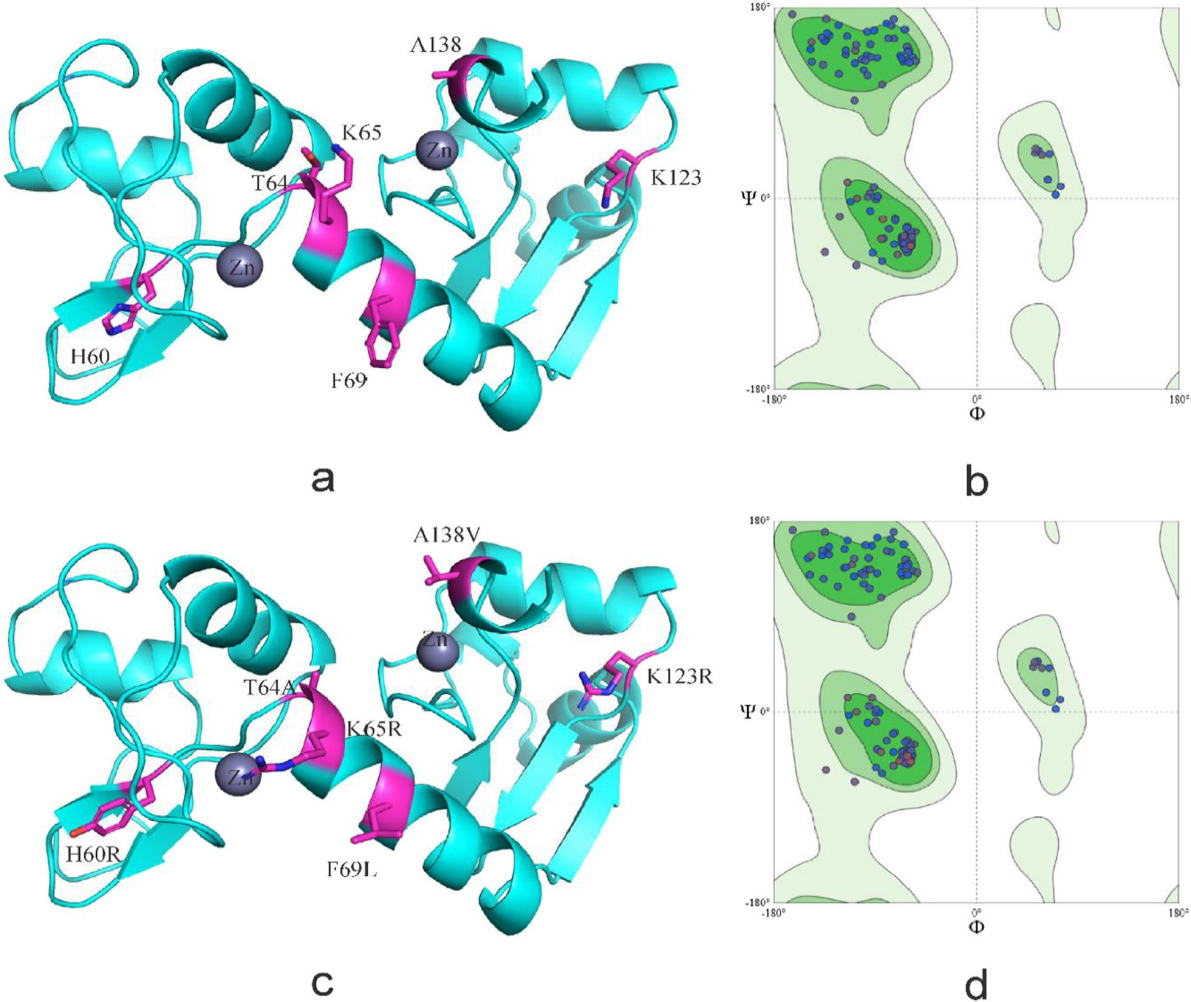
Tertiary structure of HPV-31 E6 comparing reference to the variant sequence. *Note* **a** is the homology modeling structure of HPV-31 E6 reference sequence; **b** is the larlarian diagram of HPV-31 E6 reference sequence homology modeling; **c** is homology modeling structure of HPV-31 E6 mutation sequence; d is the larchian diagram of HPV-31 E6 mutation sequence homology modeling

In HPV-31 E6, T64A, K65R, and F69L located in α-helix, T60Y located in β-sheet region, and K123R, A138V located in the coil. Amino acid substitution has no influence on the secondary and tertiary structure (Figs. 4, 5).

**Fig. 6:**
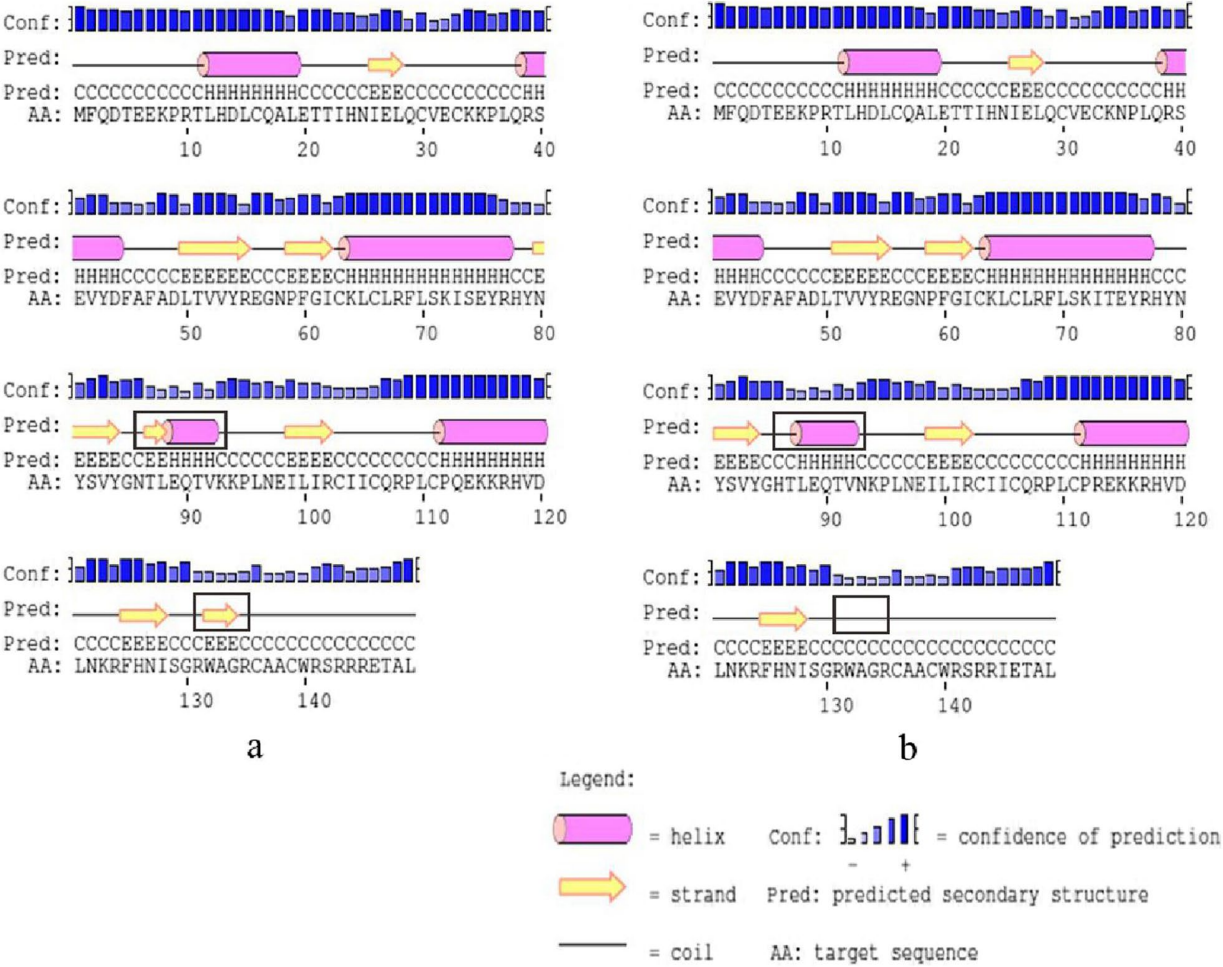
Secondary structure of HPV-33 E6 comparing reference to the variant sequence. *Note* **a** is the secondary structure pattern diagram constructed based on HPV-33 E6 reference sequence; **a** is the secondary structure pattern diagram constructed based on HPV-33 E6 mutation sequence. The black boxes are the difference areas between the reference and mutation sequence secondary structure

**Fig. 7:**
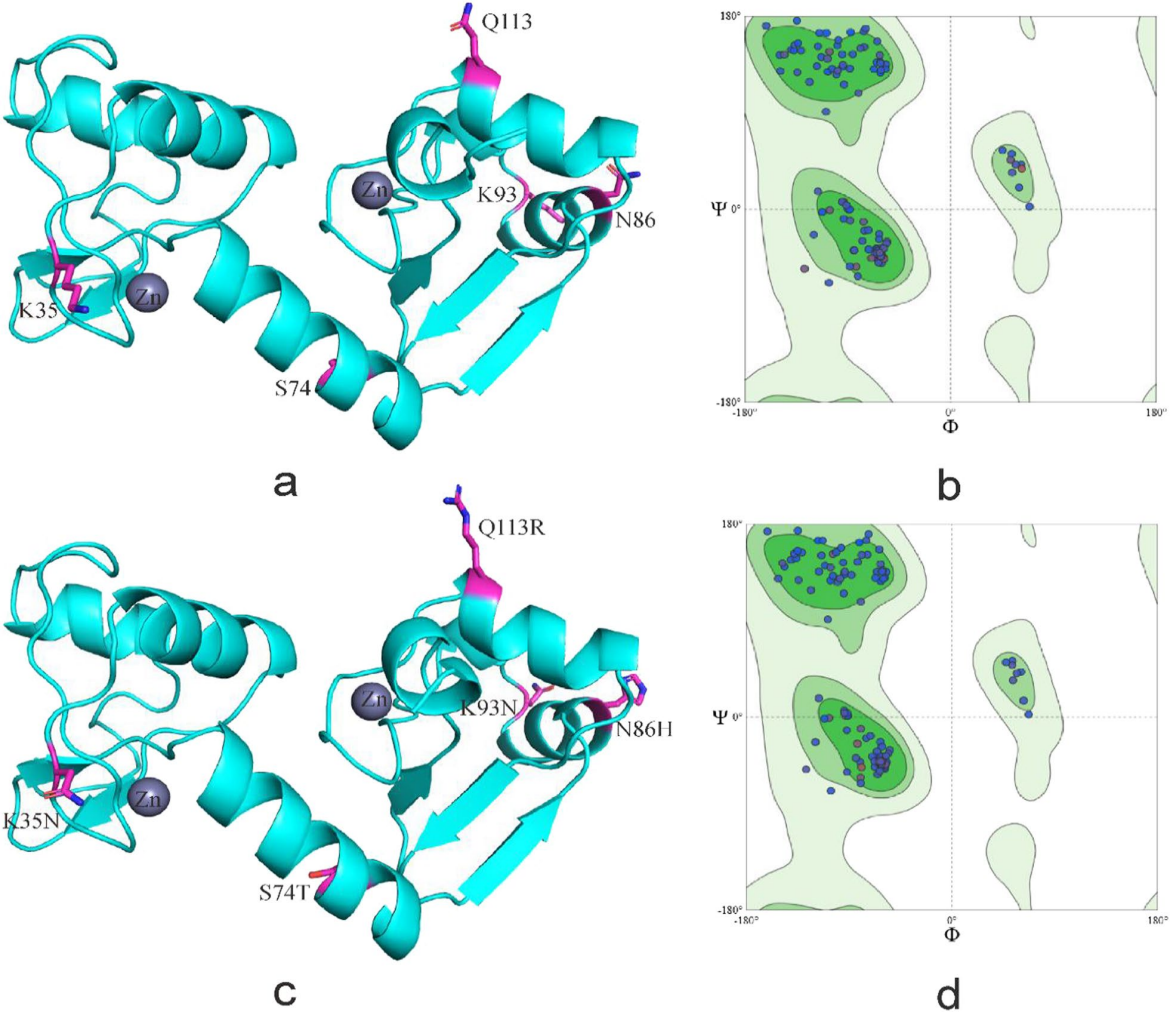
Tertiary structure of HPV-33 E6 comparing reference to the variant sequence. *Note* **a** is the homology modeling structure of HPV-33 E6 reference sequence; **b** is the larlarian diagram of HPV-33 E6 reference sequence homology modeling; **c** is homology modeling structure of HPV-33 E6 mutation sequence; d is the larlarian diagram of HPV-33 E6 mutation sequence homology modeling

S74T and Q113R located in α-helix of HPV-33 E6 protein, K93N located on the outer edge of E6 protein and near the zinc granule, the above amino acid substitutions all located in the active region of the protein. Amino acid substitution changed the number of amino acids in the α-helix and β-sheet region, as well as made the E6 protein show more contact with the environment (Figs. 6, 7).

**Fig. 8:**
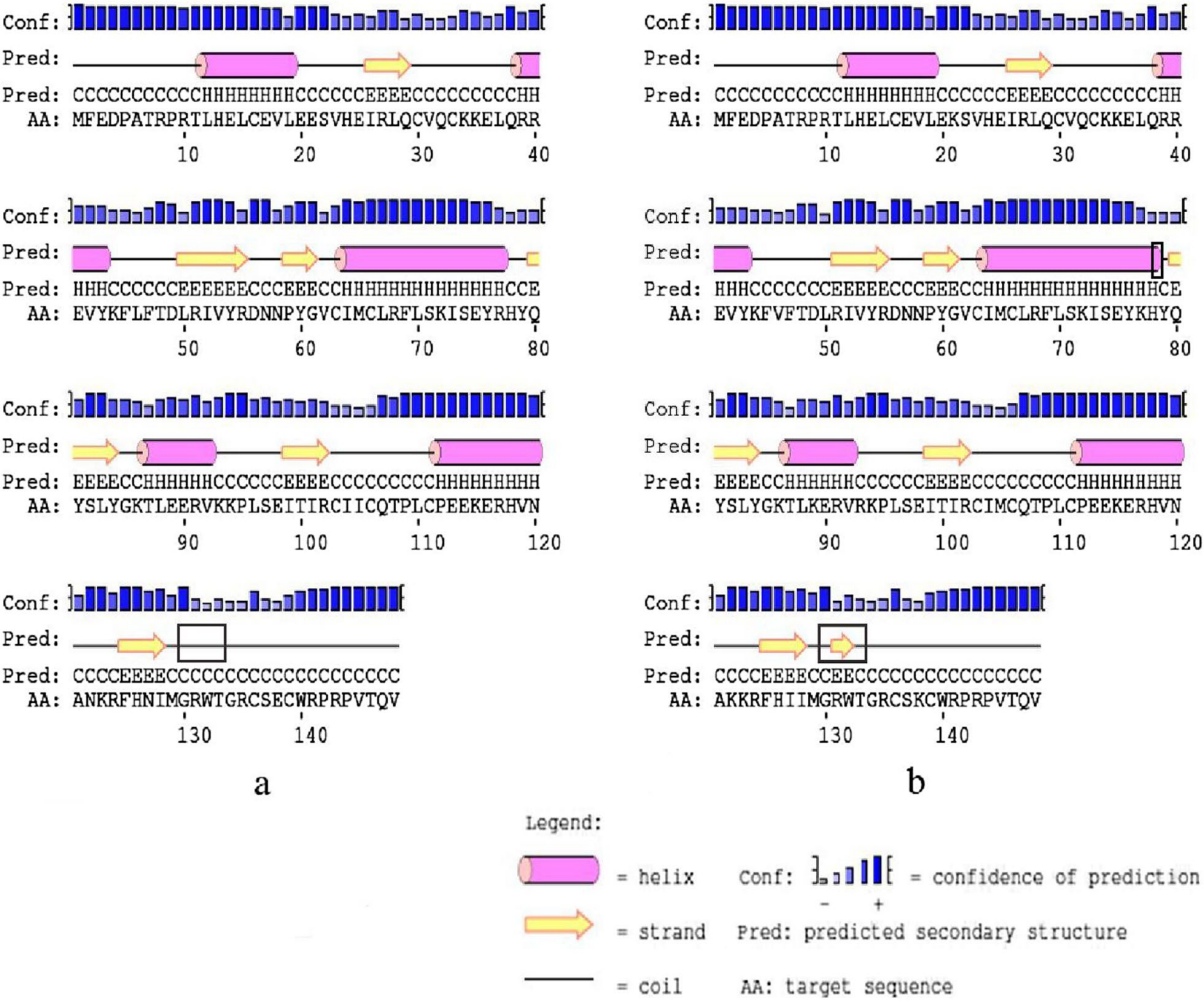
Secondary structure of HPV-52 E6 comparing reference to the variant sequence. *Note* **a** is the secondary structure pattern diagram constructed based on HPV-52 E6 reference sequence; **b** is the secondary structure pattern diagram constructed based on HPV-52 E6 mutation sequence. The black boxes are the difference areas between the reference and mutation sequence secondary structure

**Fig. 9:**
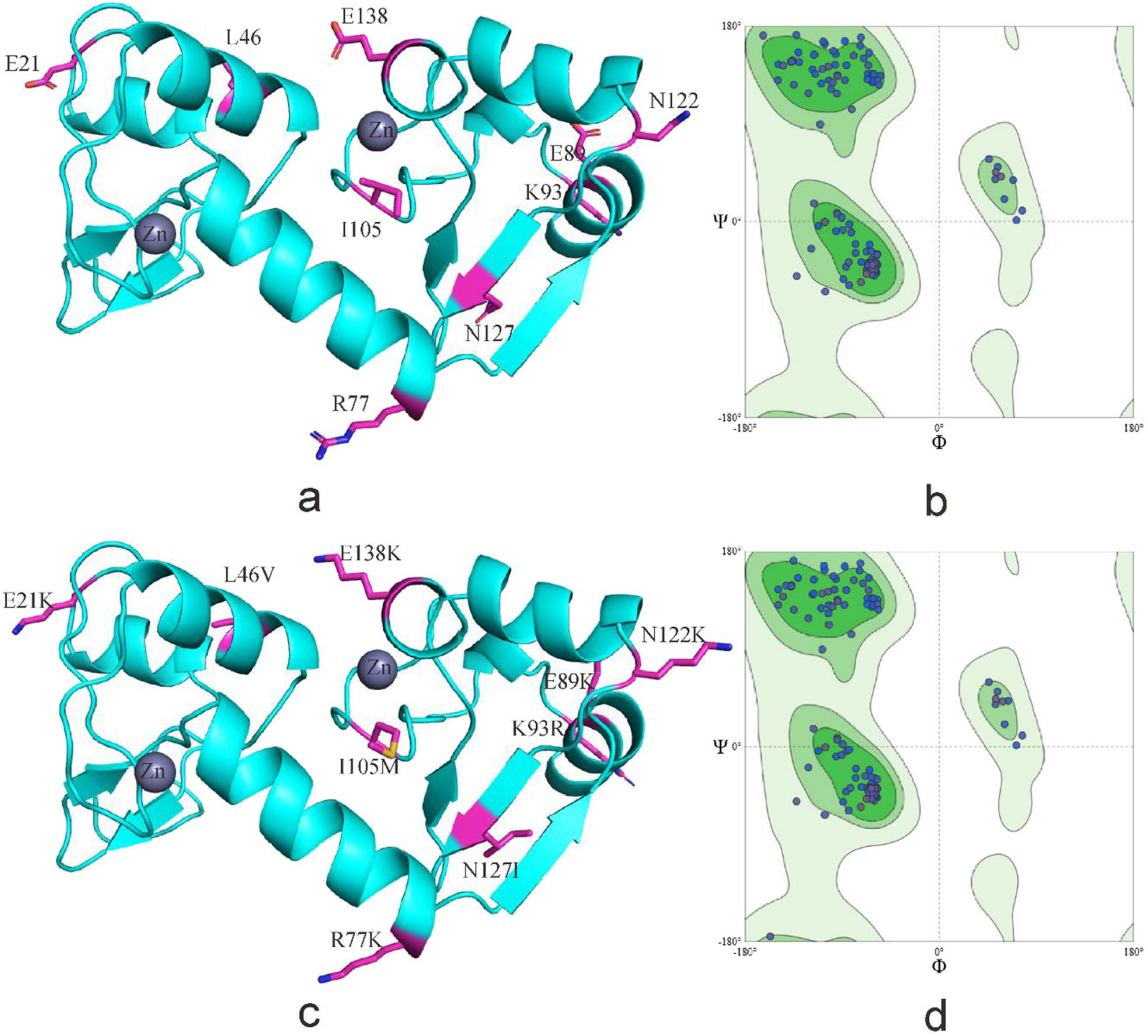
Tertiary structure of HPV-52 E6 comparing reference to the variant sequence. *Note* **a** is the homology modeling structure of HPV-52 E6 reference sequence; **b** is the larlarian diagram of HPV-52 E6 reference sequence homology modeling; **c** is homology modeling structure of HPV-52 E6 mutation sequence; d is the larlarian diagram of HPV-52 E6 mutation sequence homology modeling

R77K, E89K of HPV-52 E6 located in α-helix, N127I located in the β-sheet region, K93R situated on the outer edge of E6 protein and close to the zinc granules, all the amino acid substitutions found in the active region of the protein. Amino acid substitution increased the number of amino acids in the α-helix and β-sheet region, and the number of buried amino acids decreased (Figs. 8, 9).

**Fig. 10:**
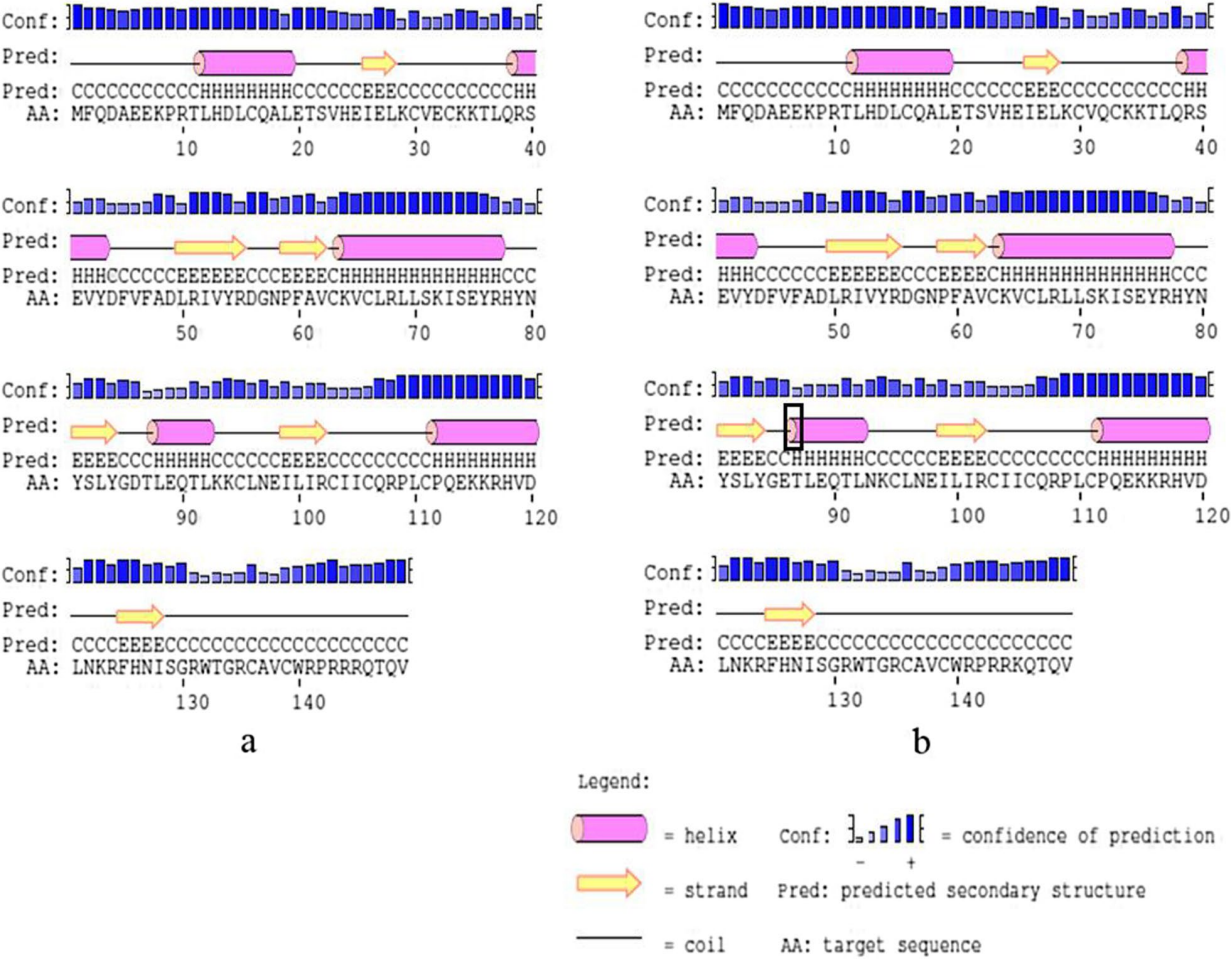
Secondary structure of HPV-58 E6 comparing reference to the variant sequence. *Note* **a** is the secondary structure pattern diagram constructed based on HPV-58 E6 reference sequence; **b** is the secondary structure pattern diagram constructed based on HPV-58 E6 mutation sequence. The black boxes are the difference areas between the reference and mutation sequence secondary structure

**Fig. 11:**
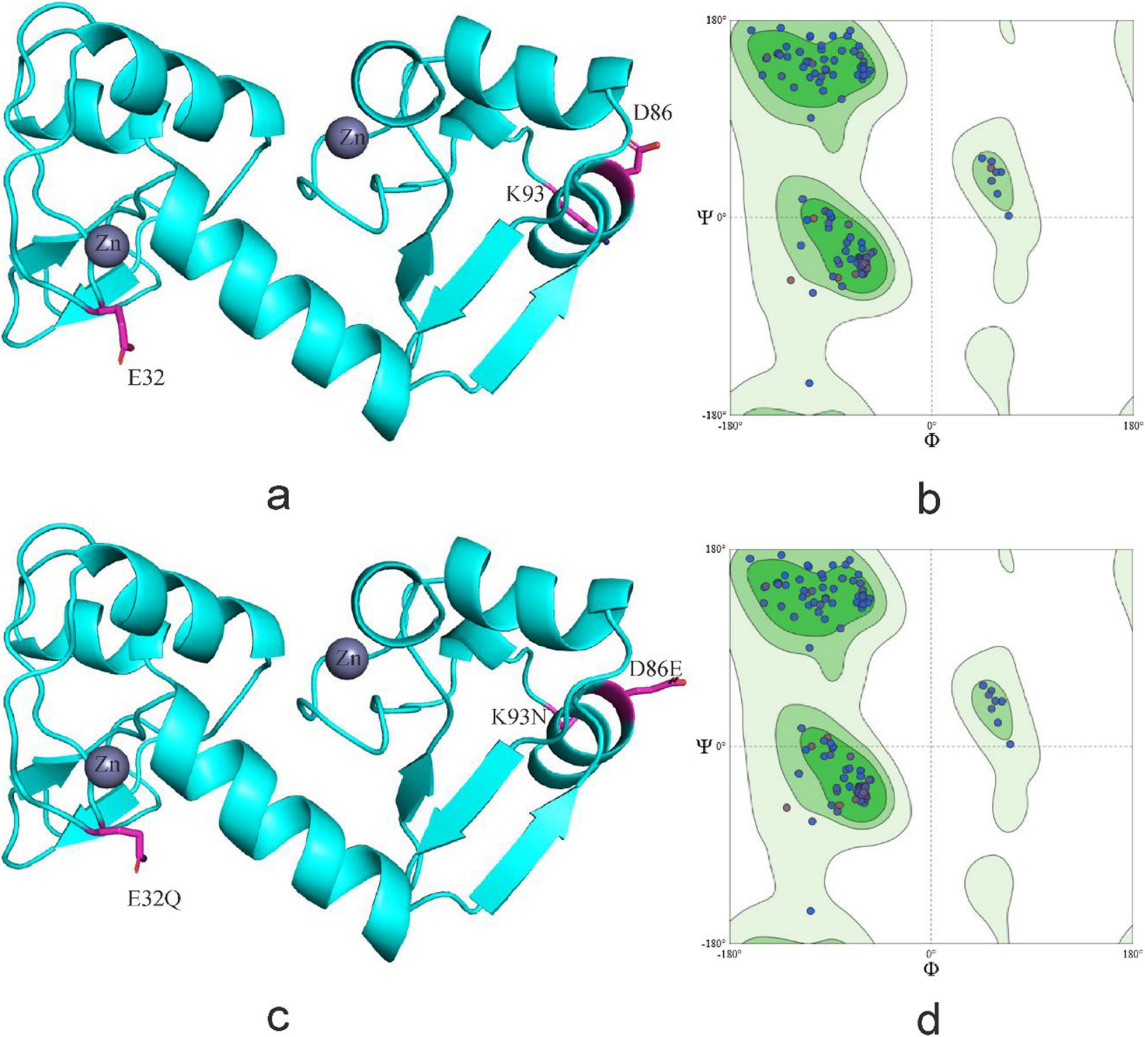
Tertiary structure of HPV-58 E6 comparing reference to the variant sequence. *Note* **a** is the homology modeling structure of HPV-58 E6 reference sequence; **b** is the larlarian diagram of HPV-58 E6 reference sequence homology modeling; **c** is homology modeling structure of HPV-58 E6 mutation sequence; **d** is the larlarian diagram of HPV-58 E6 mutation sequence homology modeling

E32Q, D86E, K93N and R145K are located in the coil of HPV-58 E6 protein, E32Q, K93N situated on the outer edge of E6 protein, and close to the zinc granule, belonging to the active region of E6 protein. Amino acid substitution increased the number of amino acids in the α-helix region and decreased the number of amino acids in the coil region (Figs. 10, 11).

### The antigen epitopes analysis of α-9 HPV E6 protein

In HPV-16 E6 reference sequence, 97 HLA-I and 25 HLA-II epitopes were selected out, and epitope prediction results of variants were different, details were shown in Additional file 1: Tables S3 and S4. M1K made epitope affinity increase; R17G, D32N, D32E, I34R, L35V, P66A, H85Y and L90V changed epitope number and affinity; E120D and R151T made new epitopes appear. The effection of amino-acid substitution on HPV-16 E6 epitopes were summarized in Table 7.

**Table 7:** Effect of Amino-acid substution on T-cell epitopes of HPV-16 E6

Substution	HPV-16 E6		
	Epitopes	Alles	Effection
M1K	1-9KHQKRTAMF	HLA-A*24:02	Better affinity
R17G	13-22QERPGKLPQL	HLA-B*40:01	Better affinity
	9-19FQDPQERPGKL	HLA-B*13:01	
	9-19FQDPQERPGKL	HLA-C*08:01	Affinity decreased
	8-17MFQDPQERPR	HLA-A*33:03	Disappear
	17-26RKLPQLCTEL	HLA-C*01:02	
	9-19FQDPQERPRKL	HLA-C*03:04	
	7-17AMFQDPQERPR	HLA-A*33:03	
D32E	26-34LQTTIHEII	HLA-B*13:01	Better affinity
	29-39TIHEIILECVY	HLA-B*15:02	Affinity decreased
	29-37TIHEIILEC	HLA-A*02:01	
	29-38TIHEIILECV	HLA-A*02:01	
	24-33TELQTTIHEI	HLA-B*40:01	New epitopes
	25-33ELQTTIHEI	HLA-B*13:01	
	32-39EIILECVY	HLA-B*15:02	
	25-33ELQTTIHEI	HLA-A*02:01	
	31-39HEIILECVY	HLA-B*15:02	
	26-33LQTTIHEI	HLA-B*13:01	
	29-39TIHDIILECVY	HLA-B*46:01	Disappear
	25-39ELQTTIHDIILECVY	HLA-DQA1*01:01/DQB1*02:01	
	24-38TELQTTIHDIILECV	HLA-DQA1*01:01/DQB1*02:01	
	25-39ELQTTIHDIILECVY	HLA-DQA1*01:01/DQB1*05:01	
	26-40LQTTIHDIILECVYC	HLA-DQA1*01:01/DQB1*02:01	
	26-40LQTTIHDIILECVYC	HLA-DQA1*01:01/DQB1*05:01	
	24-38TELQTTIHDIILECV	HLA-DQA1*01:01/DQB1*05:01	
D32N	26-34LQTTIHNII	HLA-B*13:01	Better affinity
	24-35TELQTTIHNIIL	HLA-B*40:01	
	24-33TELQTTIHNI	HLA-B*40:01	New epitopes
	27-35QTTIHNIIL	HLA-C*03:04	
	29-39TIHDIILECVY	HLA-B*15:02	Disappear
	29-37TIHDIILEC	HLA-A*02:01	
D32N	29-38TIHDIILECV	HLA-A*02:01	Disappear
	29-39TIHDIILECVY	HLA-B*46:01	
	25-39ELQTTIHDIILECVY	HLA-DQA1*01:01/DQB1*02:01	
	24-38TELQTTIHDIILECV	HLA-DQA1*01:01/DQB1*02:01	
	25-39ELQTTIHDIILECVY	HLA-DQA1*01:01/DQB1*05:01	
	26-40LQTTIHDIILECVYC	HLA-DQA1*01:01/DQB1*02:01	
	26-40LQTTIHDIILECVYC	HLA-DQA1*01:01/DQB1*05:01	
	24-38TELQTTIHDIILECV	HLA-DQA1*01:01/DQB1*05:01	
I34R	29-39TIHDIRLECVY	HLA-B*15:02	Better affinity
	29-39TIHDIRLECVY	HLA-B*46:01	Affinity decreased
	24-35TELQTTIHDIRL	HLA-B*40:01	
	25-34ELQTTIHDIR	HLA-A*33:03	New epitopes
	29-37TIHDIILEC	HLA-A*02:01	Disappear
	26-34LQTTIHDII	HLA-B*13:01	
	29-38TIHDIILECV	HLA-A*02:01	
	25-39ELQTTIHDIILECVY	HLA-DQA1*01:01/DQB1*02:01	
	24-38TELQTTIHDIILECV	HLA-DQA1*01:01/DQB1*02:01	
	25-39ELQTTIHDIILECVY	HLA-DQA1*01:01/DQB1*05:01	
	26-40LQTTIHDIILECVYC	HLA-DQA1*01:01/DQB1*02:01	
	26-40LQTTIHDIILECVYC	HLA-DQA1*01:01/DQB1*05:01	
	24-38TELQTTIHDIILECV	HLA-DQA1*01:01/DQB1*05:01	
L35V	29-39TIHDIIVECVY	HLA-B*15:02	Better affinity
	29-38TIHDIIVECV	HLA-A*02:01	
	29-39TIHDIIVECVY	HLA-B*46:01	
	29-37TIHDIIVEC	HLA-A*02:01	
	35-45VECVYCKQQLL	HLA-B*40:01	New epitopes
	24-35TELQTTIHDIIL	HLA-B*40:01	Disappear
	25-39ELQTTIHDIILECVY	HLA-DQA1*01:01/DQB1*02:01	
	24-38TELQTTIHDIILECV	HLA-DQA1*01:01/DQB1*02:01	
	25-39ELQTTIHDIILECVY	HLA-DQA1*01:01/DQB1*05:01	
	26-40LQTTIHDIILECVYC	HLA-DQA1*01:01/DQB1*02:01	
L35V	26-40LQTTIHDIILECVYC	HLA-DQA1*01:01/DQB1*05:01	Disappear
	24-38TELQTTIHDIILECV	HLA-DQA1*01:01/DQB1*05:01	
P66A	59-67IVYRDGNAY	HLA-B*15:02	Better affinity
	59-67IVYRDGNAY	HLA-B*46:01	
	59-67IVYRDGNAY	HLA-C*03:02	
	66-76AYAVCDKCLKF	HLA-A*24:02	
	61-69YRDGNAYAV	HLA-A*11:01	Affinity decreased
	59-67IVYRDGNAY	HLA-C*08:01	
	56-67DLCIVYRDGNAY	HLA-B*15:02	New epitopes
	57-67LCIVYRDGNAY	HLA-B*46:01	
	60-67VYRDGNAY	HLA-B*15:02	
	58-67CIVYRDGNAY	HLA-B*15:02	
	57-67LCIVYRDGNAY	HLA-B*15:02	
	60-69VYRDGNPYAV	HLA-A*24:02	Disappear
H85Y	85-95YYCYSLYGTTL	HLA-A*24:02	Better affinity
	77-86YSKISEYRYY	HLA-B*46:01	
	82-96EYRYYCYSLYGTTLE	HLA-DRB1*15:02	
	80-94ISEYRYYCYSLYGTT	HLA-DRB1*15:02	
	79-93KISEYRYYCYSLYGT	HLA-DRB1*15:02	
	81-95SEYRYYCYSLYGTTL	HLA-DRB1*15:02	
	78-92SKISEYRYYCYSLYG	HLA-DRB1*15:02	
	83-97YRYYCYSLYGTTLEQ	HLA-DRB1*15:02	
	77-91YSKISEYRYYCYSLY	HLA-DRB1*15:02	
	82-96EYRYYCYSLYGTTLE	HLA-DPA1*01:03/DPB1*04:01	
	83-97YRYYCYSLYGTTLEQ	HLA-DPA1*01:03/DPB1*04:01	
	77-85YSKISEYRY	HLA-C*03:02	New epitopes
	77-85YSKISEYRY	HLA-B*46:01	
	77-85YSKISEYRY	HLA-B*15:02	
	77-85YSKISEYRY	HLA-B*58:01	
	77-86YSKISEYRYY	HLA-C*03:02	
	76-85FYSKISEYRY	HLA-A*24:02	
H85Y	80-94ISEYRYYCYSLYGTT	HLA-DPA1*01:03/DPB1*04:01	New epitopes
	84-98RYYCYSLYGTTLEQQ	HLA-DPA1*01:03/DPB1*04:01	
	79-93KISEYRYYCYSLYGT	HLA-DPA1*01:03/DPB1*04:01	
	85-99YYCYSLYGTTLEQQY	HLA-DPA1*01:03/DPB1*04:01	
	84-98RYYCYSLYGTTLEQQ	HLA-DRB1*15:02	
	85-99YYCYSLYGTTLEQQY	HLA-DRB1*15:02	
	72-86KCLKFYSKISEYRYY	HLA-DRB1*12:02	
	81-95SEYRYYCYSLYGTTL	HLA-DPA1*01:03/DPB1*04:01	
	81-90SEYRYYCYSL	HLA-B*40:01	Affinity decreased
	82-90EYRYYCYSL	HLA-A*24:02	
	80-88ISEYRHYCY	HLA-C*03:02	Disappear
	77-86YSKISEYRHY	HLA-C*03:02	
	76-86FYSKISEYRHY	HLA-A*24:02	
L90V	86-95YCYSVYGTTL	HLA-C*03:04	Better affinity
	88-95YSVYGTTL	HLA-C*01:02	
	88-95YSVYGTTL	HLA-C*03:02	
	88-95YSVYGTTL	HLA-C*03:04	
	85-95HYCYSVYGTTL	HLA-A*24:02	
	89-99SVYGTTLEQQY	HLA-B*46:01	
	89-101SVYGTTLEQQYNK	HLA-A*11:01	
	86-95YCYSVYGTTL	HLA-A*24:02	
	87-95CYSVYGTTL	HLA-A*24:02	
	89-99SVYGTTLEQQY	HLA-A*11:01	
	89-99SVYGTTLEQQY	HLA-C*03:02	New epitopes
	89-97SVYGTTLEQ	HLA-A*11:01	
	89-99SVYGTTLEQQY	HLA-B*15:02	Affinity decreased
	82-90EYRHYCYSL	HLA-A*24:02	Disappear
	81-90SEYRHYCYSL	HLA-B*40:01	
	82-96EYRHYCYSLYGTTLE	HLA-DPA1*01:03/DPB1*04:01	
	83-97YRHYCYSLYGTTLEQ	HLA-DPA1*01:03/DPB1*04:01	
E120D	118-126CPDEKQRHL	HLA-C*08:01	New epitopes
E120D	118-126CPDEKQRHL	HLA-C*03:04	New epitopes
R151T	150-158STTRRETQL	HLA-C*01:02	New epitopes
	144-154MSCCRSSTTRR	HLA-A*33:03	
	150-158STTRRETQL	HLA-C*03:04	

125 HLA-I and 43 HLA-II epitopes of HPV-31 E6 reference sequence was selected out, and epitope of variants were different (Additional file 1: Tables S5, S6). H60Y, K65R changed epitope number and affinity, T64A decreased epitope number, and K123R, A138V made new epitope appear. The effection of amino-acid substitution on HPV-31 E6 epitopes were summarized in Table 8.

**Table 8:** Effect of Amino-acid substution on T-cell epitopes of HPV-31 E6

Substution	HPV-31 E6		
	Epitopes	Alles	Effection
H60Y	52-62IVYRDDTPYGV	HLA-A*02:01	Affinity decreased
	54-62YRDDTPYGV	HLA-C*08:01	
	52-60IVYRDDTPY	HLA-B*15:02	New epitopes
	51-60TIVYRDDTPY	HLA-B*15:02	
	52-60IVYRDDTPY	HLA-B*46:01	
	52-60IVYRDDTPY	HLA-C*03:02	
	57-65DTPYGVCTR	HLA-A*33:03	
	53-62VYRDDTPHGV	HLA-A*24:02	Disappear
	55-65RDDTPHGVCTK	HLA-A*11:01	
T64A	55-65RDDTPHGVCTK	HLA-A*11:01	Disappear
K65R	65-76RCLRFYSKVSEF	HLA-A*24:02	Better affinity
	57-65DTPHGVCTR	HLA-A*33:03	New epitopes
	65-79RCLRFYSKVSEFRWY	HLA-DPA1*01:03/DPB1*04:01	
	55-65RDDTPHGVCTK	HLA-A*11:01	Disappear
K123R	117-125RHLDKKRRF	HLA-A*24:02	New epitopes
A138V	132-140WTGRCIVCW	HLA-B*58:01	New epitopes

109 HLA-I and 41 HLA-II epitopes of HPV-33 E6 reference sequence was selected out, epitope of variants were different (Additional file 1: Tables S7, S8). K35N decreased epitope number and affinity; S74T, N86H, K93N and R145I changed epitope number and affinity; Q113R increased epitope affinity. The effection of amino-acid substitution on HPV-33 E6 epitopes were summarized in Table 9.

**Table 9:** Effect of Amino-acid substution on T-cell epitopes of HPV-33 E6

Substution	HPV-33 E6		
	Epitopes	Alles	Effection
K35N	35-43KPLQRSEVY	HLA-C*03:02	Disappear
	35-49NPLQRSEVYDFAFAD	HLA-DQA1*01:01/DQB1*02:01	Affinity decreased
S74T	68-76RFLSKITEY	HLA-B*46:01	Better affinity
	69-76FLSKITEY	HLA-B*46:01	
	69-77FLSKITEYR	HLA-A*33:03	
	68-77RFLSKITEYR	HLA-A*33:03	
	68-76RFLSKITEY	HLA-B*15:02	
	68-76RFLSKITEY	HLA-A*24:02	
	68-76RFLSKITEY	HLA-C*03:02	
	69-76FLSKITEY	HLA-C*03:02	
	62-76ICKLCLRFLSKISEY	HLA-DRB1*14:01	Disappear
	69-79FLSKISEYRHY	HLA-B*15:02	
	74-83SEYRHYNYSV	HLA-B*40:01	
	73-81ITEYRHYNY	HLA-C*03:02	Affinity decreased
	63-77CKLCLRFLSKITEYR	HLA-DRB1*12:02	
	64-78KLCLRFLSKITEYRH	HLA-DRB1*12:02	
	62-76ICKLCLRFLSKITEY	HLA-DRB1*12:02	
	61-75GICKLCLRFLSKITE	HLA-DRB1*12:02	
	65-79LCLRFLSKITEYRHY	HLA-DRB1*12:02	
	60-74FGICKLCLRFLSKIT	HLA-DRB1*12:02	
	66-80CLRFLSKITEYRHYN	HLA-DRB1*12:02	
	64-78KLCLRFLSKITEYRH	HLA-DRB1*14:01	
	63-77CKLCLRFLSKITEYR	HLA-DRB1*14:01	
	65-79LCLRFLSKITEYRHY	HLA-DRB1*14:01	
N86H	81-88YSVYGHTL	HLA-C*03:02	Better affinity
	81-88YSVYGHTL	HLA-C*01:02	
	81-88YSVYGHTL	HLA-C*03:04	
	81-88YSVYGHTL	HLA-C*08:01	
	80-88NYSVYGHTL	HLA-A*24:02	
	78-88HYNYSVYGHTL	HLA-A*24:02	
	79-88YNYSVYGHTL	HLA-A*24:02	
N86H	82-92SVYGHTLEQTV	HLA-A*02:01	Better affinity
	83-92VYGHTLEQTV	HLA-A*24:02	Affinity decreased
	84-92YGHTLEQTV	HLA-C*08:01	
	77-88RHYNYSVYGHTL	HLA-A*24:02	New epitopes
	82-90SVYGHTLEQ	HLA-A*11:01	
	81-88YSVYGHTL	HLA-B*46:01	
	80-88NYSVYGHTL	HLA-C*01:02	
N86H/K93N	86-94HTLEQTVNK	HLA-A*11:01	Better affinity
	85-94GHTLEQTVNK	HLA-A*11:01	
	86-94HTLEQTVNK	HLA-A*33:03	
	83-94VYGHTLEQTVNK	HLA-A*11:01	Affinity decreased
	86-96HTLEQTVNKPL	HLA-B*40:01	New epitopes
	82-93SVYGNTLEQTVK	HLA-A*11:01	Disappear
K93N	88-96LEQTVNKPL	HLA-B*40:01	Better affinity
	85-94GNTLEQTVNK	HLA-A*11:01	
	86-94NTLEQTVNK	HLA-A*11:01	
	86-94NTLEQTVNK	HLA-A*33:03	Affinity decreased
	91-102TVNKPLNEILIR	HLA-A*33:03	
	83-94VYGNTLEQTVNK	HLA-A*11:01	
	82-94SVYGNTLEQTVNK	HLA-A*11:01	
	91-99TVNKPLNEI	HLA-C*08:01	New epitopes
	91-99TVNKPLNEI	HLA-B*13:01	
	91-99TVNKPLNEI	HLA-C*03:04	
	91-99TVNKPLNEI	HLA-C*01:02	
	87-96TLEQTVNKPL	HLA-B*40:01	
	82-93SVYGNTLEQTVK	HLA-A*11:01	Disappear
Q113R	113-121REKKRHVDL	HLA-B*40:01	Better affinity
R145I	141-149RSRRIETAL	HLA-C*03:04	Better affinity
	141-149RSRRRETAL	HLA-C*01:02	Disappear

95 HLA-I and 50 HLA-II epitopes of HPV-52 E6 reference sequence was selected out, epitope of variants were different (Additional file 1: Tables S9, S10). E21K, L46V, E89K, K93R and N127I changed epitopes number and affinity; 105 M increased epitope affinity; N122K decreased epitopes number and E138K decreased epitope affinity. The effection of amino-acid substitution on HPV-52 E6 epitopes were summarized in Table 10.

**Table 10:** Effect of Amino-acid substution on T-cell epitopes of HPV-52 E6

Substution	HPV-52 E6		
	Epitopes	Alles	Effection
E21K	17-27EVLEKSVHEIR	HLA-A*33:03	Better affinity
	18-26VLEKSVHEI	HLA-B*13:01	Affinity decreased
	18-26VLEKSVHEI	HLA-A*02:01	
	19-28LEKSVHEIRL	HLA-B*40:01	
	18-26VLEKSVHEI	HLA-C*08:01	
	18-26VLEKSVHEI	HLA-C*01:02	
	19-26LEKSVHEI	HLA-B*40:01	
	10-21RTLHELCEVLEK	HLA-A*11:01	New epitopes
	20-28EESVHEIRL	HLA-B*40:01	Disappear
	82-93SLYGKTLEERVK	HLA-A*11:01	
	18-28VLEESVHEIRL	HLA-A*02:01	
	9-23PRTLHELCEVLEESV	HLA-DQA1*01:01/DQB1*02:01	
	10-24RTLHELCEVLEESVH	HLA-DQA1*01:01/DQB1*02:01	
L46V	46-55VFTDLRIVYR	HLA-A*33:03	Better affinity
	42-51VYKFVFTDLR	HLA-A*33:03	
	45-55FVFTDLRIVYR	HLA-A*33:03	
	41-51EVYKFVFTDLR	HLA-A*33:03	
	45-54FVFTDLRIVY	HLA-B*46:01	
	40-47REVYKFVF	HLA-B*40:01	
	46-54VFTDLRIVY	HLA-B*46:01	
	42-50VYKFVFTDL	HLA-A*24:02	
	45-54FVFTDLRIVY	HLA-C*03:02	
	46-54VFTDLRIVY	HLA-C*03:02	
	45-54FVFTDLRIVY	HLA-B*15:02	Affinity decreased
	44-52KFVFTDLRI	HLA-A*24:02	
	45-53FVFTDLRIV	HLA-A*02:01	
	41-55EVYKFVFTDLRIVYR	HLA-DPA1*01:03/DPB1*04:01	
	39-53RREVYKFVFTDLRIV	HLA-DPA1*01:03/DPB1*04:01	
	43-57YKFVFTDLRIVYRDN	HLA-DPA1*01:03/DPB1*04:01	
	38-52QRREVYKFVFTDLRI	HLA-DPA1*01:03/DPB1*04:01	
	37-51LQRREVYKFVFTDLR	HLA-DPA1*01:03/DPB1*04:01	
L46V	44-58KFVFTDLRIVYRDNN	HLA-DPA1*01:03/DPB1*04:01	Affinity decreased
	36-50ELQRREVYKFVFTDL	HLA-DPA1*01:03/DPB1*04:01	
	41-55EVYKFVFTDLRIVYR	HLA-DRB1*14:01	
	42-56VYKFVFTDLRIVYRD	HLA-DRB1*14:01	
	43-57YKFVFTDLRIVYRDN	HLA-DRB1*14:01	
	40-54REVYKFVFTDLRIVY	HLA-DRB1*14:01	
	41-55EVYKFVFTDLRIVYR	HLA-DRB1*12:02	
	46-54VFTDLRIVY	HLA-A*24:02	New epitopes
	40-50REVYKFVFTDL	HLA-B*40:01	
	45-53FVFTDLRIV	HLA-B*46:01	
	45-53FVFTDLRIV	HLA-C*03:02	
	45-53FVFTDLRIV	HLA-C*03:04	
	40-54REVYKFLFTDLRIVY	HLA-DQA1*01:01/DQB1*05:01	Disappear
	39-53RREVYKFLFTDLRIV	HLA-DQA1*01:01/DQB1*05:01	
	41-55EVYKFLFTDLRIVYR	HLA-DQA1*01:01/DQB1*05:01	
	42-56VYKFLFTDLRIVYRD	HLA-DQA1*01:01/DQB1*05:01	
	42-56VYKFLFTDLRIVYRD	HLA-DRB1*12:02	
	40-54REVYKFLFTDLRIVY	HLA-DQA1*01:01/DQB1*02:01	
	41-55EVYKFLFTDLRIVYR	HLA-DQA1*01:01/DQB1*02:01	
	40-54REVYKFLFTDLRIVY	HLA-DRB1*12:02	
	44-58KFLFTDLRIVYRDNN	HLA-DRB1*14:01	
	43-57YKFLFTDLRIVYRDN	HLA-DRB1*12:02	
E89K	82-92SLYGKTLKERV	HLA-A*02:01	Affinity decreased
	86-94KTLKERVKK	HLA-A*11:01	
	85-94GKTLKERVKK	HLA-A*11:01	
	82-93SLYGKTLKERVK	HLA-A*11:01	
	41-55EVYKFVFTDLRIVYR	HLA-DPA1*01:03/DPB1*04:01	
	39-53RREVYKFVFTDLRIV	HLA-DPA1*01:03/DPB1*04:01	
	43-57YKFVFTDLRIVYRDN	HLA-DPA1*01:03/DPB1*04:01	
	38-52QRREVYKFVFTDLRI	HLA-DPA1*01:03/DPB1*04:01	
	37-51LQRREVYKFVFTDLR	HLA-DPA1*01:03/DPB1*04:01	
E89K	44-58KFVFTDLRIVYRDNN	HLA-DPA1*01:03/DPB1*04:01	Affinity decreased
	36-50ELQRREVYKFVFTDL	HLA-DPA1*01:03/DPB1*04:01	
	42-56VYKFVFTDLRIVYRD	HLA-DRB1*14:01	
	41-55EVYKFVFTDLRIVYR	HLA-DRB1*14:01	
	43-57YKFVFTDLRIVYRDN	HLA-DRB1*14:01	
	40-54REVYKFVFTDLRIVY	HLA-DRB1*14:01	
	41-55EVYKFVFTDLRIVYR	HLA-DRB1*12:02	
	82-91SLYGKTLKER	HLA-A*11:01	Better affinity
	82-91SLYGKTLKER	HLA-A*33:03	
	75-89EYRHYQYSLYGKTLK	HLA-DPA1*01:03/DPB1*04:01	
	81-89YSLYGKTLK	HLA-A*11:01	New epitopes
	79-89YQYSLYGKTLK	HLA-A*11:01	
	80-89QYSLYGKTLK	HLA-A*11:01	
	82-94SLYGKTLEERVKK	HLA-A*11:01	Disappear
	84-94YGKTLEERVKK	HLA-A*11:01	
	40-54REVYKFLFTDLRIVY	HLA-DQA1*01:01/DQB1*05:01	
	39-53RREVYKFLFTDLRIV	HLA-DQA1*01:01/DQB1*05:01	
	41-55EVYKFLFTDLRIVYR	HLA-DQA1*01:01/DQB1*05:01	
	42-56VYKFLFTDLRIVYRD	HLA-DQA1*01:01/DQB1*05:01	
	42-56VYKFLFTDLRIVYRD	HLA-DRB1*12:02	
	40-54REVYKFLFTDLRIVY	HLA-DQA1*01:01/DQB1*02:01	
	41-55EVYKFLFTDLRIVYR	HLA-DQA1*01:01/DQB1*02:01	
	40-54REVYKFLFTDLRIVY	HLA-DRB1*12:02	
	44-58KFLFTDLRIVYRDNN	HLA-DRB1*14:01	
	43-57YKFLFTDLRIVYRDN	HLA-DRB1*12:02	
K93R	85-94GKTLEERVRK	HLA-A*11:01	Affinity decreased
	82-94SLYGKTLEERVRK	HLA-A*11:01	
	84-94YGKTLEERVRK	HLA-A*11:01	
	88-96LEERVRKPL	HLA-B*40:01	
	82-93SLYGKTLEERVR	HLA-A*33:03	New epitopes
	91-99RVKKPLSEI	HLA-B*13:01	Disappear
K93R	82-93SLYGKTLEERVK	HLA-A*11:01	Disappear
I105M	97-105SEITIRCIM	HLA-B*40:01	Better affinity
N122K	120-128NANKRFHNI	HLA-C*08:01	Disappear
	120-128NANKRFHNI	HLA-C*03:04	
N127I	127-135IIMGRWTGR	HLA-A*33:03	Affinity decreased
	124-132RFHIIMGRW	HLA-A*24:02	
	126-135HIIMGRWTGR	HLA-A*33:03	Better affinity
	125-135FHIIMGRWTGR	HLA-A*33:03	
	120-128NANKRFHII	HLA-C*03:04	
	120-128NANKRFHII	HLA-C*08:01	
	120-131NANKRFHIIMGR	HLA-A*33:03	New epitopes
	121-135ANKRFHIIMGRWTGR	HLA-DRB1*12:02	
	123-137KRFHIIMGRWTGRCS	HLA-DRB1*12:02	
	122-136NKRFHIIMGRWTGRC	HLA-DRB1*12:02	
	120-134NANKRFHIIMGRWTG	HLA-DRB1*12:02	
	119-133VNANKRFHIIMGRWT	HLA-DRB1*12:02	
	124-138RFHIIMGRWTGRCSE	HLA-DRB1*12:02	
	124-132RFHNIMGRW	HLA-B*58:01	Disappear
E138K	132-140WTGRCSKCW	HLA-B*58:01	Affinity decreased

113 HLA-I and 44 HLA-II epitopes of HPV-58 E6 reference sequence was selected out, epitope of variants were different (Additional file 1: Tables S11, S12). D86E, K93N changed epitopes number and affinity, and R145K changed HLA-I epitope. The effection of amino-acid substitution on HPV-58 E6 epitopes were summarized in Table 11.

**Table 11:** Effect of Amino-acid substution on T-cell epitopes of HPV-58 E6

Substution	HPV-58 E6		
	Epitopes	Alles	Effection
D86E	81-88YSLYGETL	HLA-C*03:04	better affinity
	84-92YGETLEQTL	HLA-C*08:01	
	81-88YSLYGETL	HLA-C*08:01	
	80-88NYSLYGETL	HLA-A*24:02	
	79-88YNYSLYGETL	HLA-A*24:02	
	78-88HYNYSLYGETL	HLA-A*24:02	
	83-92LYGETLEQTL	HLA-A*24:02	Affinity decreased
	82-91SLYGETLEQT	HLA-A*02:01	
	84-92YGETLEQTL	HLA-C*03:04	
	81-88YSLYGETL	HLA-C*01:02	
	82-92SLYGETLEQTL	HLA-B*13:01	
	85-92GETLEQTL	HLA-B*40:01	New epitopes
	81-88YSLYGETL	HLA-C*03:02	
	84-92YGETLEQTL	HLA-C*01:02	
	84-92YGDTLEQTL	HLA-C*03:02	Disappear
D86E/K93N	85-94GETLEQTLNK	HLA-A*11:01	Better affinity
	86-94ETLEQTLNK	HLA-A*11:01	
	82-94SLYGETLEQTLNK	HLA-A*11:01	Affinity decreased
	84-94YGETLEQTLNK	HLA-A*11:01	
	85-96GETLEQTLNKCL	HLA-B*40:01	New epitopes
	86-94ETLEQTLNK	HLA-A*33:03	
	82-93SLYGDTLEQTLK	HLA-A*11:01	Disappear
K93N	88-96LEQTLNKCL	HLA-B*40:01	Better affinity
	85-94GDTLEQTLNK	HLA-A*11:01	
	84-94YGDTLEQTLNK	HLA-A*11:01	Affinity decreased
	82-94SLYGDTLEQTLNK	HLA-A*11:01	
	91-99TLNKCLNEI	HLA-A*02:01	New epitopes
	82-93SLYGDTLEQTLK	HLA-A*11:01	Disappear
R145K	137-145AVCWRPRRK	HLA-A*11:01	New epitopes
	137-145AVCWRPRRR	HLA-A*33:03	Disappear

## Discussion

Cervical cancer is the second major malignant tumor in women in childbearing age and seriously threatens women's health. HR-HPV persistent infection is closely related to the occurrence and development of cervical cancer and other malignant diseases. α-9 HPV is almost all carcinogenic and associated with 75% cervical cancers. Sichuan is a multi-ethnic mixed residence area with a high prevalence rate of α-9 HPV. From 2012 to 2017, α-9 HPV positive samples accounted for 53.68% of all HPV positive samples and 73.22% of high-risk HPV positive samples, showing an increasing trend.

α-9 genus HPV is highly prevalent and pathogenic; its carcinogenicity is mainly realized through E6 oncoproteins guided by the *E6* gene through triggering the immortalization of infected cells. As an early gene of the virus, HPV *E6* has a high mutation risk. In Sichuan, 21, 13, 8, 21, 8 nucleotide mutations were detected in HPV-16, HPV-31, HPV-33, HPV-52, HPV-58 *E6* respectively, and among them, 17, 6, 6, 13, 4 were non-synonymous mutation.

Gene non-synonymous mutations change the amino acid composition, and structure of the protein, as well as the functions of protein, are mainly realized by its structures. HPV E6 consists of one N-terminal (residues 1–36), one C-terminal (residues 147–158) and two Zinc fingers (residues 37–73 and 110–146, CxxC-(29x)-CxxC) three domains. The two Zinc finger binding domains form a deep pocket, which can mediate the most important tumor suppressor protein p53 ubiquitination degradation by binding to the "LXXLL" sequence of E6AP protein [24, 25]. 145–149 were PDZ domain-containing combined region that was the target of E6 protein for cellular transformation and the carboxy-terminal half being principally involved in p53 binding [26]. K93N of HPV-33 E6, K93R of HPV-52 E6, and K93N of HPV-58 E6 are located at the outer edge of E6 protein and near the zinc granule [27]. The N86H, R145I of HPV-33 E6 and D86E, R145K of HPV-58 E6 occurred in the same positions; K93N of HPV-33 E6, K93R of HPV-52 E6 and K93N of HPV-58 E6 all located in the 93rd of the E6 protein; those amino acid substitutions located in protein active region, can cause the E6 terminal and the trend of the carboxyl end structure disorder. Those protein conformational changes may lead to the differences in their ability to bind to the host p53 protein and other potential proteins, thus affecting the pathogenicity of α-9 HPV [29].

Positive selection sites make the gene frequency of the corresponding amino acid increasingly stable and enhance the species' adaptability to the environment [28]. According to the calculation, the positive selection site of HPV-16 E6 was D32E (128/250); HPV-33 E6 were K35N (42/216), K93N (42/216), R145I (33/216); HPV-52 E6 was K93R (252/288); HPV-58 E6 were K93N (111/405), R145K (16/405); These positive selection sites all belong to its high frequency non-synonymous mutation, suggesting that these positive selection sites, which contribute to the adaptation of α-9 HPV E6, have been widely spread.

HPV E6 protein plays a key role in cervical cancer development. During HPV infection, the immune system will treat E6 protein as an antigen presentation to eliminate HPV infection and reduce the occurrence risk of HPV-related diseases with the help of body immunity [30]. Some specific mutations in HPV E6 may lead to the differences in the infection ability and pathogenicity of the virus. Positive selection sites of HPV-16 E6 D32E, D32N located in protein outer edge and next to the zinc granules; 6 HLA-II epitopes disappeared due to D32E/D32N; In Japan, D32E has been confirmed to be associated with the development of cervical cancer [31]; T-cell antigen epitopes affinity reduced due to D32E, D32N, that may lead to the persistent infection of virus and promote the development of cervical cancer. Positive selection sites K35N and K93N of HPV-33 E6 are close to the zinc granules, while R145I located in the E6 PDZ binding domain; K35N and R145I made 35-43KPLQRSEVY for HLA-C*03:02 and 141-149RSRRRETAL for HLA-C*01:02 disappear respectively, K93N changed the epitope number and affinity; Above three positive selection sites of HPV-33 E6 located in E6 protein active region, affect the protein conformation, function and reduced the immunogenicity of the peptide containing the above sites to a certain amount. The positive selection site K93R of HPV-52 E6 changed the epitope number and decreased the affinity of excellent epitopes. K93N and R145I of HPV-58 E6 reduced the affinity of excellent HLA-I antigen epitopes. Those positive selection sites reduced the immunogenicity of E6 overall, which may make HPV-infected cells more difficult to be detected by the immune system, and enhance HPV adaptability to the environment. No positive selection site was selected out in HPV-31 E6, and the high-frequency non-synonymous mutation sites enhanced the affinity and number of E6 epitopes, which may relate to its extremely low prevalence.

Studies have found that mutations affect the efficiency of HPV vaccine [32], the protein structure and antigen epitope bioinformatics prediction method were introduced to analyze the influence of HPV *E6* mutation on protein conformational and immunogenicity. We discussed the relationship between protein structure, positive selection site, antigen epitope and pathogenicity of α-9 HPV E6 protein in Sichuan was discussed for the first time. Amino acid substitution in positive selection sites may affect the virus infection efficiency, immunogenicity, and pathogenicity by altering their T-cell epitopes affinity to improve the survival ability of α-9 HPV as well as an adaptation to evolution. These results help explore the relationship between HPV E6 polymorphism and HPV affection capacity and its action mechanism to improve the therapeutic vaccine of α-9 HPV in Sichuan regions of China.

## Conclusion

α-9 HPV is extremely prevalent in Sichuan, China. The positive selection site K93N of HPV-33 E6, K93R of HPV-52 E6 and K93N of HPV-58 E6 all occurred in the 93rd amino acid of E6 protein. N86H, R145I (positive selection site) of HPV-33 E6 and D86E, R145K (positive selection site) of HPV-58 E6 occurred in the same location of E6. α-9 HPV E6 positive selection sites that adaptive to the environment D32E, K35N, K93N, R145I, K93R, R145K have been widely spread, they all located in the E6 protein active region and altered their protein structure, as well as overall reduce the immunogenicity of the E6 protein, so that HPV infected cells are more difficult to be detected by the immune system and enhance the adaptability of α-9 HPV to the environment.

E6 mutations in positive selection sites may affect the virus infection efficiency, immunogenicity by altering their protein structure, epitopes affinity to improve the survival ability of HPV.

## Supplementary Information

**Additional file 1: Table S1.** Primers and PCR condition used for the molecular characterization of α-9 HPV E6. **Table S2.** Average frequency of HLA-I and HLA-II alleles (> 5%) across the Chinese population. **Table S3.** The HLA-I predicted epitopes of HPV-16 E6. **Table S4.** The HLA-II predicted epitopes of HPV-16 E6. **Table S5.** The HLA-I predicted epitopes of HPV-31 E6. **Table S6.** The HLA-II predicted epitopes of HPV-31 E6. **Table S7.** The HLA-I predicted epitopes of HPV-33 E6. **Table S8.** The HLA-II predicted epitopes of HPV-33 E6. **Table S9.** The HLA-I predicted epitopes of HPV-52 E6. **Table S10.** The HLA-II predicted epitopes of HPV-52 E6. **Table S11.** The HLA-I predicted epitopes of HPV-58 E6. **Table S12.** The HLA-II predicted epitopes of HPV-58 E6.

## Data Availability

All data generated or analyzed during this study are included in this article and GeneBank.

## Abbreviations

CC: Cervical cancer
HPV: Human Papillomavirus
HLA: Human leukocyte antigen
CTL: Cytotoxic T lymphocytes
Th: Helper T lymphocytes
NCBI: National Center for Biotechnology Information
dN: Non-synonymous mutation rate
dS: Synonymous mutation rate
dbMHC: Major histocompatibility complex database
PR: Percentile rank
HR-HPV: High-risk human papilloma virus
MEGA 6: Molecular Evolutionary Genetics Analysis version 6.0
