# Supplementary material for: The polymorphism analysis and epitope predicted of Alphapapillomavirus 9 E6 in Sichuan, China
Source: Virol J. 2022 Jan 20;19:14. doi: 10.1186/s12985-021-01728-4 (PMC8772103; doi:10.1186/s12985-021-01728-4)
Supplement: Supplementary file 1 — Additional file 1: Table S1. Primers and PCR condition used for the molecular characterization of α-9 HPV E6. Table S2. Average frequency of HLA-I and HLA-II alleles (> 5%) across the Chinese population. Table S3. The HLA-I predicted epitopes of HPV-16 E6. Table S4. The HLA-II predicted epitopes of HPV-16 E6. Table S5. The HLA-I predicted epitopes of HPV-31 E6. Table S6. The HLA-II predicted epitopes of HPV-31 E6. Table S7. The HLA-I predicted epitopes of HPV-33 E6. Table S8. The HLA-II predicted epitopes of HPV-33 E6. Table S9. The HLA-I predicted epitopes of HPV-52 E6. Table S10. The HLA-II predicted epitopes of HPV-52 E6. Table S11. The HLA-I predicted epitopes of HPV-58 E6. Table S12. The HLA-II predicted epitopes of HPV-58 E6. [file 12985_2021_1728_MOESM1_ESM.pdf]

The polymorphism analysis and epitope predicted of α-9 HPV E6 in Sichuan

Jiaoyu He[1,2,3], Shiyu Ma[2,3], Qiufu Li[2,3], Yiran Liu[2,3], Yanru Cui[2,3], Jianying Peng[2,3], Zhuang Ju[2,3], Yunfan Shi[2,3], Xia Wei[2,3], Xianping Ding[2,3]*.

1 Phamaceutical sciences and Chinese medicine college, southwest university, Chongqing 400000.

2 Chongqing Nanchuan biotechnology research institute, Bio-resource Research and Utilization Joint Key Laboratory of Sichuan and Chongqing, Sichuan and Chongqing, P.R.China.

3 Key Laboratory of Bio-Resources and Eco-Environment of Ministry of Education, College of Life Sciences, Sichuan University, Chengdu 610065, Sichuan, P.R.China.

Address for Correspondence: Institute of Medical Genetics, College of Life Sciences, Sichuan University, Chengdu 610064, China.

* Corresponding author:

Institute of Medical Genetics, College of Life Sciences, Sichuan University, Chengdu 610064, China.

E-mail: brainding@scu.edu.cn

Telephone: 86-028-85413096

Fax: 86-028-85415895

Email address:

Jiaoyu He: 1061355567@qq.com; Shiyu Ma: 895686227@qq.com;

Qiufu Li: lqf1192069072@126.com; Yiran Liu: 532154290@qq.com;

Yanru Cui: 512927123@qq.com; Jianying Peng: 2271644005@qq.com;

Zhuang Ju: 929882573@qq.com; Yunfan Shi: 2811829836@qq.com;

Xia Wei: 531197860@qq.com; Xianping Ding: brainding@scu.edu.cn.

Table S1. Primers and PCR condition used for the molecular characterization of α-9 HPV E6
Table S2. Average frequency of HLA-I and HLA-II alleles (>5%) across the Chinese population
Table S3. The HLA-I predicted epitopes of HPV-16 E6
Table S4. The HLA-II predicted epitopes of HPV-16 E6
Table S5. The HLA-I predicted epitopes of HPV-31 E6
Table S6. The HLA-II predicted epitopes of HPV-31 E6
Table S7. The HLA-I predicted epitopes of HPV-33 E6
Table S8. The HLA-II predicted epitopes of HPV-33 E6
Table S9. The HLA-I predicted epitopes of HPV-52 E6
Table S10. The HLA-II predicted epitopes of HPV-52 E6
Table S11. The HLA-I predicted epitopes of HPV-58 E6
Table S12. The HLA-II predicted epitopes of HPV-58 E6

Table S1. Primers and PCR condition used for the molecular characterization of α-9 HPV E6

Ref NO.	Primer name	Sequence 5′ to 3′	PCR condition
NC001526	HPV16 E6F	ACTAAGGGCGTAACCG	95℃Pre-denaturation 5 min, 94℃ denaturation 45 s,
	HPV16 E6R	AATGGGCTCTGTCCG	55℃ annealing 1 min, 72℃ extension 1 min.
J04353	HPV31 E7F	GAACCGAAAACG TTGGTATATAAAGCACT	95℃ pre- denaturation 5 min, 94℃ denaturation 45
	HPV31 E7R	AGGTTGCAAATCTAACACATAGTCTTGCAAC	s, 55℃ annealing 50 s, 72℃ extension 1 min.
M12732.1	HPV33 E6F	AAGTAGGGTGTAACCG	95℃ pre-denaturation 5 min, 94℃ denaturation 45 s,
	HPV33 E6R	AACGCCATGAGAGAGG	58℃ annealing 45 s, 72℃ extension 1 min.
NC001592	HPV52 E7F	GAATCGGTGCATGAAATAAG	95℃ pre-denaturation 5 min, 94℃ denaturation 45 s,
	HPV52 E7R	TTGTTTCAGGT TGCAGATC	55℃ annealing 1 min, 72℃ extension 1 min.
D90400	HPV58 E6F	CGAAAACGGTCTGACCGAAA	95℃ pre-denaturation 5 min, 94℃ denaturation 45 s,
	HPV58 E6R	AAACAACCCAACGTCAAGAG	58℃annealing 45 s, and 72℃ extension 1 min.

Note: Ref NO. means the number of HPV reference sequence in NCBI.

Table S2. Average frequency of HLA-I and HLA-II alleles (>5%) across the Chinese population

Alleles	Average frequency	Alleles	Average frequency	Alleles	Average frequency
HLA-A*11:01	27.7%	HLA-A*33:03	11.5%	DPB1*04:01	17.1%
HLA-A*24:02	17.2%	HLA-B*58:01	8.9%	DRB1*14:01	13.4%
HLA-C*01:02	16.9%	HLA-C*03:02	8.7%	DRB1*12:02	11.4%
HLA-B*40:01	14.9%	HLA-B*13:01	8.2%	DQB1*05:01	10.9%
HLA-C*03:04	12.8%	HLA-B*15:02	7.1%	DQB1*02:01	9.3%
HLA-C*08:01	12.6%	HLA-A*02:01	5.3%	DRB1*15:02	5.6%
HLA-B*46:01	11.5%				

Table S3. The HLA-I predicted epitopes of HPV-16 E6

HPV-16 E6 Reference			HPV-16 E6 Variant		
epitopes	allele	score	epitopes	allele	score
93-101TTLEQQYNK	HLA-A*11:01	0.01	59-67IVYRDGNAY	HLA-B*15:02	0.02
18-26KLPQLCTEL	HLA-C*01:02	0.02	59-67IVYRDGNAY	HLA-B*46:01	0.02
59-67IVYRDGNPY	HLA-B*15:02	0.04	88-95YSVYGTTL	HLA-C*03:04	0.04
89-99SLYGTTLEQQY	HLA-B*15:02	0.06	59-67IVYRDGNAY	HLA-C*03:02	0.05
76-84FYSKISEYR	HLA-A*33:03	0.06	89-99SVYGTTLEQQY	HLA-B*15:02	0.09
59-67IVYRDGNPY	HLA-B*46:01	0.06	89-99SVYGTTLEQQY	HLA-B*46:01	0.15
52-61FAFRDLCIVY	HLA-B*46:01	0.07	66-76AYAVCDKCLKF	HLA-A*24:02	0.16
88-95YSLYGTTL	HLA-C*03:04	0.07	88-95YSVYGTTL	HLA-C*08:01	0.18
92-101GTTLEQQYNK	HLA-A*11:01	0.09	89-101SVYGTTLEQQYNK	HLA-A*11:01	0.18
59-67IVYRDGNPY	HLA-C*03:02	0.11	77-85YSKISEYRY	HLA-C*03:02	0.21
8-17MFQDPQERPR	HLA-A*33:03	0.13	26-34LQTTIHEII	HLA-B*13:01	0.22
134-142NIRGRWTGR	HLA-A*33:03	0.16	87-95CYSVYGTTL	HLA-A*24:02	0.22
95-103LEQQYNKPL	HLA-B*40:01	0.18	88-95YSVYGTTL	HLA-C*03:02	0.22
97-107QQYNKPLCDLL	HLA-B*13:01	0.18	89-99SVYGTTLEQQY	HLA-A*11:01	0.22
88-95YSLYGTTL	HLA-C*08:01	0.18	13-22QERPGKLPQL	HLA-B*40:01	0.23
53-62AFRDLCIVYR	HLA-A*33:03	0.2	77-85YSKISEYRY	HLA-B*46:01	0.24
42-50QQLLRREVY	HLA-B*15:02	0.2	88-95YSVYGTTL	HLA-C*01:02	0.3
75-83KFYSKISEY	HLA-B*15:02	0.21	26-34LQTTIHNII	HLA-B*13:01	0.34
52-61FAFRDLCIVY	HLA-C*03:02	0.22	29-39TIHDIRLECVY	HLA-B*15:02	0.34
87-95CYSLYGTTL	HLA-A*24:02	0.23	29-39TIHDIIVECVY	HLA-B*15:02	0.36
91-99YGTTLEQQY	HLA-C*03:02	0.24	77-85YSKISEYRY	HLA-B*15:02	0.36
89-99SLYGTTLEQQY	HLA-B*46:01	0.25	118-126CPDEKQRHL	HLA-C*08:01	0.38
75-83KFYSKISEY	HLA-B*46:01	0.26	77-85YSKISEYRY	HLA-B*58:01	0.4
48-55EVYDFAFR	HLA-A*33:03	0.28	86-95YCYSVYGTTL	HLA-A*24:02	0.4
49-57VYDFAFRDL	HLA-A*24:02	0.28	57-67LCIVYRDGNAY	HLA-B*15:02	0.41
66-76PYAVCDKCLKF	HLA-A*24:02	0.28	89-99SVYGTTLEQQY	HLA-C*03:02	0.41
75-83KFYSKISEY	HLA-C*03:02	0.3	29-38TIHDIIVECV	HLA-A*02:01	0.46
75-84KFYSKISEYR	HLA-A*33:03	0.31	24-33TELQTTIHEI	HLA-B*40:01	0.51
88-95YSLYGTTL	HLA-C*03:02	0.31	85-95YYCYSLYGTTL	HLA-A*24:02	0.51
81-90SEYRHYCYSL	HLA-B*40:01	0.37	1-9KHQKRTAMF	HLA-A*24:02	0.52
9-19FQDPQERPRKL	HLA-C*08:01	0.37	9-19FQDPQERPGKL	HLA-C*08:01	0.52
53-61AFRDLCIVY	HLA-B*15:02	0.38	25-34ELQTTIHDIR	HLA-A*33:03	0.52
52-61FAFRDLCIVY	HLA-B*15:02	0.38	56-67DLCIVYRDGNAY	HLA-B*15:02	0.52
29-39TIHDIILECVY	HLA-B*15:02	0.39	29-39TIHDIIVECVY	HLA-B*46:01	0.55
61-69YRDGNPYAV	HLA-C*08:01	0.39	77-86YSKISEYRYY	HLA-B*46:01	0.55
67-76YAVCDKCLKF	HLA-B*46:01	0.4	25-33ELQTTIHEI	HLA-B*13:01	0.59
88-95YSLYGTTL	HLA-C*01:02	0.4	81-90SEYRYYCYSL	HLA-B*40:01	0.62
133-142HNIRGRWTGR	HLA-A*33:03	0.41	29-39TIHEIILECVY	HLA-B*15:02	0.63
18-26KLPQLCTEL	HLA-A*02:01	0.41	77-86YSKISEYRYY	HLA-C*03:02	0.64
86-95YCYSLYGTTL	HLA-A*24:02	0.41	24-35TELQTTIHNIIL	HLA-B*40:01	0.65

Continued table 1: Table S3. The HLA-I predicted epitopes of HPV-16 E6

HPV-16 E6 Reference			HPV-16 E6 Variant		
epitopes	allele	score	epitopes	allele	score
91-99YGTTLEQQY	HLA-B*46:01	0.43	29-39TIHDIRLECVY	HLA-B*46:01	0.69
98-107QYNKPLCDLL	HLA-A*24:02	0.44	150-158STTRRETQL	HLA-C*01:02	0.69
97-106QQYNKPLCDL	HLA-B*13:01	0.45	24-35TELQTTIHEIIL	HLA-B*40:01	0.7
38-45VYCKQQLL	HLA-A*24:02	0.45	29-37TIHDIIVEC	HLA-A*02:01	0.7
124-132RHLDKKQRF	HLA-A*24:02	0.46	61-69YRDGNAYAV	HLA-C*08:01	0.72
131-139RFHNIRGRW	HLA-A*24:02	0.46	58-67CIVYRDGNAY	HLA-B*15:02	0.72
98-106QYNKPLCDL	HLA-A*24:02	0.47	9-19FQDPQERPGKL	HLA-B*13:01	0.73
9-19FQDPQERPRKL	HLA-B*13:01	0.48	24-35TELQTTIHDIRL	HLA-B*40:01	0.73
67-76YAVCDKCLKF	HLA-C*03:02	0.49	76-85FYSKISEYRY	HLA-A*24:02	0.73
76-83FYSKISEY	HLA-C*03:02	0.51	86-95YCYSVYGTTL	HLA-C*03:04	0.75
17-26RKLPQLCTEL	HLA-C*01:02	0.52	85-95HYCYSVYGTTL	HLA-A*24:02	0.81
1-9MHQKRTAMF	HLA-A*24:02	0.54	150-158STTRRETQL	HLA-C*03:04	0.81
76-83FYSKISEY	HLA-B*15:02	0.55	32-39EIILECVY	HLA-B*15:02	0.83
75-83KFYSKISEY	HLA-A*24:02	0.55	29-37TIHEIILEC	HLA-A*02:01	0.88
98-108QYNKPLCDLLI	HLA-A*24:02	0.55	25-33ELQTTIHEI	HLA-A*02:01	0.89
7-15AMFQDPQER	HLA-A*11:01	0.56	29-38TIHEIILECV	HLA-A*02:01	0.89
91-99YGTTLEQQY	HLA-B*15:02	0.56	24-33TELQTTIHNI	HLA-B*40:01	0.89
26-34LQTTIHDII	HLA-B*13:01	0.56	89-97SVYGTTLEQ	HLA-A*11:01	0.89
76-83FYSKISEY	HLA-B*46:01	0.56	144-154MSCCRSSTTRR	HLA-A*33:03	0.89
29-38TIHDIILECV	HLA-A*02:01	0.57	31-39HEIILECVY	HLA-B*15:02	0.91
82-90EYRHYCYSL	HLA-A*24:02	0.57	35-45VECVYCKQQLL	HLA-B*40:01	0.93
6-15TAMFQDPQER	HLA-A*33:03	0.58	57-67LCIVYRDGNAY	HLA-B*46:01	0.93
49-57VYDFAFRDL	HLA-C*01:02	0.58	60-67VYRDGNAY	HLA-B*15:02	0.97
77-86YSKISEYRHY	HLA-B*46:01	0.6	82-90EYRYYCYSL	HLA-A*24:02	0.98
51-62DFAFRDLCIVYR	HLA-A*33:03	0.61	26-33LQTTIHEI	HLA-B*13:01	0.99
29-39TIHDIILECVY	HLA-B*46:01	0.61	27-35QTTIHNIIL	HLA-C*03:04	0.99
91-101YGTTLEQQYNK	HLA-A*11:01	0.63	59-67IVYRDGNAY	HLA-A*11:01	0.99
7-15AMFQDPQER	HLA-A*33:03	0.64	118-126CPDEKQRHL	HLA-C*03:04	1
39-47YCKQQLLRR	HLA-A*33:03	0.64			
53-61AFRDLCIVY	HLA-B*46:01	0.64			
89-101SLYGTTLEQQYNK	HLA-A*11:01	0.67			
13-22QERPRKLPQL	HLA-B*40:01	0.68			
24-35TELQTTIHDIIL	HLA-B*40:01	0.7			
52-60FAFRDLCIV	HLA-C*03:04	0.71			
5-15RTAMFQDPQER	HLA-A*11:01	0.73			
77-86YSKISEYRHY	HLA-C*03:02	0.76			
41-49KQQLLRREV	HLA-B*13:01	0.78			
29-37TIHDIILEC	HLA-A*02:01	0.78			
38-46VYCKQQLLR	HLA-A*33:03	0.8			
47-57REVYDFAFRDL	HLA-B*40:01	0.8			

Continued table 2: Table S3. The HLA-I predicted epitopes of HPV-16 E6

HPV-16 E6 Reference			HPV-16 E6 Variant		
epitopes	allele	score	epitopes	allele	score
47-54REVYDFAF	HLA-B*40:01	0.81			
74-83LKFYSKISEY	HLA-B*15:02	0.83			
85-95HYCYSLYGTTL	HLA-A*24:02	0.83			
86-95YCYSLYGTTL	HLA-C*03:04	0.84			
8-15MFQDPQER	HLA-A*33:03	0.85			
93-101TTLEQQYNK	HLA-A*33:03	0.88			
49-57VYDFAFRDL	HLA-C*08:01	0.9			
76-86FYSKISEYRHY	HLA-A*24:02	0.92			
59-67IVYRDGNPY	HLA-A*11:01	0.93			
80-88ISEYRHYCY	HLA-C*03:02	0.93			
68-76AVCDKCLKF	HLA-B*46:01	0.94			
77-84YSKISEYR	HLA-A*33:03	0.95			
131-139RFHNIRGRW	HLA-B*58:01	0.96			
89-99SLYGTTLEQQY	HLA-A*11:01	0.98			
60-69VYRDGNPYAV	HLA-A*24:02	0.99			
9-19FQDPQERPRKL	HLA-C*03:04	0.99			
7-17AMFQDPQERPR	HLA-A*33:03	1			

Table S4. The HLA-II predicted epitopes of HPV-16 E6

HPV-16 E6 Reference			HPV-16 E6 Variant		
epitopes	allele	score	epitopes	allele	score
47-61REVYDFAFRDLCIVY	HLA-DQA1*01:01/DQB1*05:01	1.1	82-96EYRYYCYSLYGTTLE	HLA-DRB1*15:02	0.85
46-60RREVYDFAFRDLCIV	HLA-DQA1*01:01/DQB1*05:01	1.1	80-94ISEYRYYCYSLYGTT	HLA-DRB1*15:02	0.85
45-59LRREVYDFAFRDLCI	HLA-DQA1*01:01/DQB1*05:01	1.4	79-93KISEYRYYCYSLYGT	HLA-DRB1*15:02	0.85
48-62EVYDFAFRDLCIVYR	HLA-DQA1*01:01/DQB1*05:01	1.7	81-95SEYRYYCYSLYGTTL	HLA-DRB1*15:02	0.85
49-63VYDFAFRDLCIVYRD	HLA-DQA1*01:01/DQB1*05:01	1.8	78-92SKISEYRYYCYSLYG	HLA-DRB1*15:02	0.85
82-96EYRHYCYSLYGTTLE	HLA-DRB1*15:02	2.1	83-97YRYYCYSLYGTTLEQ	HLA-DRB1*15:02	0.85
80-94ISEYRHYCYSLYGTT	HLA-DRB1*15:02	2.1	77-91YSKISEYRYYCYSLY	HLA-DRB1*15:02	0.85
79-93KISEYRHYCYSLYGT	HLA-DRB1*15:02	2.1	82-96EYRYYCYSLYGTTLE	HLA-DPA1*01:03/DPB1*04:01	1.6
81-95SEYRHYCYSLYGTTL	HLA-DRB1*15:02	2.1	81-95SEYRYYCYSLYGTTL	HLA-DPA1*01:03/DPB1*04:01	1.7
78-92SKISEYRHYCYSLYG	HLA-DRB1*15:02	2.1	83-97YRYYCYSLYGTTLEQ	HLA-DPA1*01:03/DPB1*04:01	1.8
83-97YRHYCYSLYGTTLEQ	HLA-DRB1*15:02	2.1	80-94ISEYRYYCYSLYGTT	HLA-DRB1*15:02	2.1
77-91YSKISEYRHYCYSLY	HLA-DRB1*15:02	2.1	84-98RYYCYSLYGTTLEQQ	HLA-DRB1*15:02	2.1
45-59LRREVYDFAFRDLCI	HLA-DQA1*01:01/DQB1*02:01	2.9	79-93KISEYRYYCYSLYGT	HLA-DRB1*15:02	2.1
25-39ELQTTIHDIILECVY	HLA-DQA1*01:01/DQB1*02:01	3	85-99YYCYSLYGTTLEQQY	HLA-DRB1*15:02	2.1
46-60RREVYDFAFRDLCIV	HLA-DQA1*01:01/DQB1*02:01	3.1	84-98RYYCYSLYGTTLEQQ	HLA-DRB1*15:02	2.1
47-61REVYDFAFRDLCIVY	HLA-DQA1*01:01/DQB1*02:01	3.3	85-99YYCYSLYGTTLEQQY	HLA-DRB1*15:02	2.1
46-60RREVYDFAFRDLCIV	HLA-DPA1*01:03/DPB1*04:01	3.8	82-96EYRHYCYSVYGTTLE	HLA-DRB1*15:02	2.1
24-38TELQTTIHDIILECV	HLA-DQA1*01:01/DQB1*02:01	4	80-94ISEYRHYCYSVYGTT	HLA-DPA1*01:03/DPB1*04:01	2.9
25-39ELQTTIHDIILECVY	HLA-DQA1*01:01/DQB1*05:01	4.1	79-93KISEYRHYCYSVYGT	HLA-DPA1*01:03/DPB1*04:01	3.2
47-61REVYDFAFRDLCIVY	HLA-DPA1*01:03/DPB1*04:01	4.2	81-95SEYRHYCYSVYGTTL	HLA-DPA1*01:03/DPB1*04:01	3.7
26-40LQTTIHDIILECVYC	HLA-DQA1*01:01/DQB1*02:01	4.5	78-92SKISEYRHYCYSVYG	HLA-DPA1*01:03/DPB1*04:01	4
82-96EYRHYCYSLYGTTLE	HLA-DPA1*01:03/DPB1*04:01	4.6	83-97YRHYCYSVYGTTLEQ	HLA-DRB1*15:02	4.1
83-97YRHYCYSLYGTTLEQ	HLA-DPA1*01:03/DPB1*04:01	4.6	77-91YSKISEYRHYCYSVY	HLA-DRB1*15:02	4.1
26-40LQTTIHDIILECVYC	HLA-DQA1*01:01/DQB1*05:01	4.7	72-86KCLKFYSKISEYRYY	HLA-DRB1*12:02	4.7
24-38TELQTTIHDIILECV	HLA-DQA1*01:01/DQB1*05:01	4.8			

Table S5. The HLA-I predicted epitopes of HPV-31 E6

HPV-31 E6 Reference			HPV-31 E6 Variant		
epitopes	allele	score	epitopes	allele	score
82-90SVYGTTLEK	HLA-A*11:01	0.01	52-60IVYRDDTPY	HLA-B*15:02	0.08
70-78YSKVSEFRW	HLA-B*58:01	0.01	52-60IVYRDDTPY	HLA-B*46:01	0.1
86-94TTLEKLTNK	HLA-A*11:01	0.01	52-60IVYRDDTPY	HLA-C*03:02	0.22
72-80KVSEFRWYR	HLA-A*33:03	0.01	57-65DTPYGVCTR	HLA-A*33:03	0.49
45-54FAFTDLTIVY	HLA-B*46:01	0.01	52-62IVYRDDTPYGV	HLA-A*02:01	0.51
68-76RFYSKVSEF	HLA-A*24:02	0.02	51-60TIVYRDDTPY	HLA-B*15:02	0.59
83-91VYGTTLEKL	HLA-A*24:02	0.03	132-140WTGRCIVCW	HLA-B*58:01	0.69
35-43GQLTETEVL	HLA-B*13:01	0.03	54-62YRDDTPYGV	HLA-C*08:01	0.7
80-90RYSVYGTTLEK	HLA-A*11:01	0.04	65-76RCLRFYSKVSEF	HLA-A*24:02	0.81
45-54FAFTDLTIVY	HLA-C*03:02	0.04	117-125RHLDKKRRF	HLA-A*24:02	0.86
81-88YSVYGTTL	HLA-C*03:04	0.04	57-65DTPYGVCTR	HLA-A*33:03	0.38
80-88RYSVYGTTL	HLA-A*24:02	0.05			
11-19KLHELSSAL	HLA-A*02:01	0.06			
71-80SKVSEFRWYR	HLA-A*33:03	0.06			
13-21HELSSALEI	HLA-B*13:01	0.06			
11-19KLHELSSAL	HLA-B*13:01	0.06			
13-21HELSSALEI	HLA-B*40:01	0.07			
45-54FAFTDLTIVY	HLA-B*15:02	0.07			
72-80KVSEFRWYR	HLA-A*11:01	0.08			
85-94GTTLEKLTNK	HLA-A*11:01	0.09			
81-90YSVYGTTLEK	HLA-A*11:01	0.09			
69-77FYSKVSEFR	HLA-A*33:03	0.09			
69-76FYSKVSEF	HLA-A*24:02	0.13			
69-78FYSKVSEFRW	HLA-A*24:02	0.14			
47-54FTDLTIVY	HLA-C*03:02	0.14			
70-80YSKVSEFRWYR	HLA-A*33:03	0.15			
46-54AFTDLTIVY	HLA-B*15:02	0.15			
82-94SVYGTTLEKLTNK	HLA-A*11:01	0.17			
1-10MFKNPAERPR	HLA-A*33:03	0.17			
81-88YSVYGTTL	HLA-C*08:01	0.18			
45-53FAFTDLTIV	HLA-C*03:04	0.19			
11-19KLHELSSAL	HLA-C*01:02	0.19			
45-53FAFTDLTIV	HLA-C*08:01	0.19			
46-55AFTDLTIVYR	HLA-A*33:03	0.21			
69-78FYSKVSEFRW	HLA-B*58:01	0.22			
47-55FTDLTIVYR	HLA-A*33:03	0.22			
127-135NIGGRWTGR	HLA-A*33:03	0.22			
81-88YSVYGTTL	HLA-C*03:02	0.22			
68-78RFYSKVSEFRW	HLA-B*58:01	0.24			
19-28LEIPYDELRL	HLA-B*40:01	0.24			

Continued table 1: Table S5. The HLA-I predicted epitopes of HPV-31 E6

HPV-31 E6 Reference			HPV-31 E6 Variant		
epitopes	allele	score	epitopes	allele	score
126-135HNIGGRWTGR	HLA-A*33:03	0.28			
19-26LEIPYDEL	HLA-B*40:01	0.28			
81-88YSVYGTTL	HLA-C*01:02	0.3			
68-77RFYSKVSEFR	HLA-A*33:03	0.31			
52-62IVYRDDTPHGV	HLA-A*02:01	0.32			
68-78RFYSKVSEFRW	HLA-A*24:02	0.32			
124-132RFHNIGGRW	HLA-A*24:02	0.33			
45-53FAFTDLTIV	HLA-C*03:02	0.34			
68-76RFYSKVSEF	HLA-C*03:02	0.34			
68-76RFYSKVSEF	HLA-B*46:01	0.34			
25-32ELRLNCVY	HLA-B*15:02	0.35			
47-54FTDLTIVY	HLA-B*46:01	0.36			
11-19KLHELSSAL	HLA-B*46:01	0.37			
17-27SALEIPYDELR	HLA-A*33:03	0.38			
80-88RYSVYGTTL	HLA-C*01:02	0.38			
68-76RFYSKVSEF	HLA-B*15:02	0.4			
79-88YRYSVYGTTL	HLA-A*24:02	0.4			
69-80FYSKVSEFRWYR	HLA-A*33:03	0.41			
42-50VLDFAFTDL	HLA-C*08:01	0.41			
82-91SVYGTTLEKL	HLA-A*11:01	0.42			
11-19KLHELSSAL	HLA-C*03:04	0.43			
14-23ELSSALEIPY	HLA-B*15:02	0.45			
6-15AERPRKLHEL	HLA-B*40:01	0.46			
45-53FAFTDLTIV	HLA-B*46:01	0.46			
15-23LSSALEIPY	HLA-C*03:02	0.48			
79-90YRYSVYGTTLEK	HLA-A*11:01	0.5			
45-52FAFTDLTI	HLA-C*03:04	0.5			
46-54AFTDLTIVY	HLA-C*03:02	0.51			
54-62YRDDTPHGV	HLA-C*08:01	0.51			
47-54FTDLTIVY	HLA-B*15:02	0.52			
46-54AFTDLTIVY	HLA-B*46:01	0.52			
46-55AFTDLTIVYR	HLA-A*11:01	0.53			
45-52FAFTDLTI	HLA-C*08:01	0.53			
67-76LRFYSKVSEF	HLA-A*24:02	0.54			
20-28EIPYDELRL	HLA-C*01:02	0.54			
44-55DFAFTDLTIVYR	HLA-A*33:03	0.55			
47-55FTDLTIVYR	HLA-A*11:01	0.56			
77-90RWYRYSVYGTTLEK	HLA-A*11:01	0.56			
11-19KLHELSSAL	HLA-B*15:02	0.57			
21-32IPYDELRLNCVY	HLA-B*15:02	0.57			

Continued table 2: Table S5. The HLA-I predicted epitopes of HPV-31 E6

HPV-31 E6 Reference			HPV-31 E6 Variant		
epitopes	allele	score	epitopes	allele	score
86-94TTLEKLTNK	HLA-A*33:03	0.58			
47-54FTDLTIVY	HLA-C*08:01	0.58			
84-94YGTTLEKLTNK	HLA-A*11:01	0.59			
68-76RFYSKVSEF	HLA-C*01:02	0.6			
100-108LIRCITCQR	HLA-A*33:03	0.61			
35-43GQLTETEVL	HLA-B*40:01	0.61			
15-23LSSALEIPY	HLA-B*46:01	0.61			
45-55FAFTDLTIVYR	HLA-A*33:03	0.62			
34-43KGQLTETEVL	HLA-B*13:01	0.62			
73-80VSEFRWYR	HLA-A*33:03	0.63			
1-8MFKNPAER	HLA-A*33:03	0.63			
38-47TETEVLDFAF	HLA-B*40:01	0.63			
17-26SALEIPYDEL	HLA-C*03:04	0.63			
15-23LSSALEIPY	HLA-B*58:01	0.64			
17-26SALEIPYDEL	HLA-C*08:01	0.64			
71-80SKVSEFRWYR	HLA-A*11:01	0.67			
15-23LSSALEIPY	HLA-B*15:02	0.67			
124-132RFHNIGGRW	HLA-B*58:01	0.69			
68-80RFYSKVSEFRWYR	HLA-A*33:03	0.71			
69-76FYSKVSEF	HLA-C*03:02	0.71			
132-140WTGRCIACW	HLA-B*58:01	0.72			
71-78SKVSEFRW	HLA-B*58:01	0.72			
82-91SVYGTTLEKL	HLA-A*24:02	0.73			
68-76RFYSKVSEF	HLA-B*13:01	0.74			
11-19KLHELSSAL	HLA-C*03:02	0.75			
11-19KLHELSSAL	HLA-C*08:01	0.75			
82-90SVYGTTLEK	HLA-A*33:03	0.78			
42-50VLDFAFTDL	HLA-C*01:02	0.78			
67-78LRFYSKVSEFRW	HLA-B*58:01	0.79			
35-45GQLTETEVLDF	HLA-B*13:01	0.8			
82-91SVYGTTLEKL	HLA-C*03:04	0.81			
11-21KLHELSSALEI	HLA-A*02:01	0.82			
81-91YSVYGTTLEKL	HLA-A*24:02	0.84			
55-65RDDTPHGVCTK	HLA-A*11:01	0.87			
6-19AERPRKLHELSSAL	HLA-B*40:01	0.89			
4-12NPAERPRKL	HLA-C*08:01	0.91			
45-53FAFTDLTIV	HLA-A*02:01	0.93			
82-91SVYGTTLEKL	HLA-B*46:01	0.94			
11-21KLHELSSALEI	HLA-B*40:01	0.94			
46-54AFTDLTIVY	HLA-A*24:02	0.94			

Continued table 3: Table S5. The HLA-I predicted epitopes of HPV-31 E6

HPV-31 E6 Reference			HPV-31 E6 Variant		
epitopes	allele	score	epitopes	allele	score
4-12NPAERPRKL	HLA-C*03:04	0.94			
53-62VYRDDTPHGV	HLA-A*24:02	0.96			
42-50VLDFAFTDL	HLA-A*02:01	0.97			
45-52FAFTDLTI	HLA-C*03:02	0.97			
65-76KCLRFYSKVSEF	HLA-A*24:02	1			

Table S6. The HLA-II predicted epitopes of HPV-31 E6

HPV-31 E6 Reference			HPV-31 E6 Variant		
epitopes	allele	score	epitopes	allele	score
40-54TEVLDFAFTDLTIVY	HLA-DQA1*01:01/DQB1*02:01	0.58	65-79RCLRFYS KVSEFRWY	HLA-DPA1*01:03/ DPB1*04:01	5.0
41-55EVLDFAFTDLTIVYR	HLA-DQA1*01:01/DQB1*02:01	0.69			
39-53ETEVLDFAFTDLTIV	HLA-DQA1*01:01/DQB1*02:01	0.71			
77-91RWYRYSVYGTTLEKL	HLA-DPA1*01:03/DPB1*04:01	0.95			
41-55EVLDFAFTDLTIVYR	HLA-DPA1*01:03/DPB1*04:01	1.1			
74-88SEFRWYRYSVYGTTL	HLA-DPA1*01:03/DPB1*04:01	1.2			
40-54TEVLDFAFTDLTIVY	HLA-DPA1*01:03/DPB1*04:01	1.2			
78-92WYRYSVYGTTLEKLT	HLA-DPA1*01:03/DPB1*04:01	1.2			
75-89EFRWYRYSVYGTTLE	HLA-DPA1*01:03/DPB1*04:01	1.3			
42-56VLDFAFTDLTIVYRD	HLA-DQA1*01:01/DQB1*02:01	1.4			
38-52TETEVLDFAFTDLTI	HLA-DQA1*01:01/DQB1*02:01	1.5			
76-90FRWYRYSVYGTTLEK	HLA-DPA1*01:03/DPB1*04:01	1.6			
42-56VLDFAFTDLTIVYRD	HLA-DPA1*01:03/DPB1*04:01	1.7			
73-87VSEFRWYRYSVYGTT	HLA-DPA1*01:03/DPB1*04:01	1.7			
39-53ETEVLDFAFTDLTIV	HLA-DQA1*01:01/DQB1*05:01	2.1			
40-54TEVLDFAFTDLTIVY	HLA-DQA1*01:01/DQB1*05:01	2.1			
39-53ETEVLDFAFTDLTIV	HLA-DPA1*01:03/DPB1*04:01	2.2			
36-50QLTETEVLDFAFTDL	HLA-DQA1*01:01/DQB1*02:01	2.4			
37-51LTETEVLDFAFTDLT	HLA-DQA1*01:01/DQB1*02:01	2.5			
75-89EFRWYRYSVYGTTLE	HLA-DRB1*15:02	2.6			
76-90FRWYRYSVYGTTLEK	HLA-DRB1*15:02	2.6			
72-86KVSEFRWYRYSVYGT	HLA-DRB1*15:02	2.6			
43-57LDFAFTDLTIVYRDD	HLA-DQA1*01:01/DQB1*02:01	2.6			
74-88SEFRWYRYSVYGTTL	HLA-DRB1*15:02	2.6			
71-85SKVSEFRWYRYSVYG	HLA-DRB1*15:02	2.6			
73-87VSEFRWYRYSVYGTT	HLA-DRB1*15:02	2.6			
79-93YRYSVYGTTLEKLTN	HLA-DPA1*01:03/DPB1*04:01	2.6			
70-84YSKVSEFRWYRYSVY	HLA-DRB1*15:02	2.6			
41-55EVLDFAFTDLTIVYR	HLA-DQA1*01:01/DQB1*05:01	2.7			
72-86KVSEFRWYRYSVYGT	HLA-DPA1*01:03/DPB1*04:01	2.7			

Continued table 1: Table S6. The HLA-II predicted epitopes of HPV-31 E6

HPV-31 E6 Reference			HPV-31 E6 Variant		
epitopes	allele	score	epitopes	allele	score
43-57LDFAFTDLTIVYRDD	HLA-DPA1*01:03/DPB1*04:01	3.1			
77-91RWYRYSVYGTTLEKL	HLA-DRB1*15:02	3.3			
78-92WYRYSVYGTTLEKLT	HLA-DRB1*15:02	3.3			
35-49GQLTETEVLDFAFTD	HLA-DQA1*01:01/DQB1*02:01	3.4			
70-84YSKVSEFRWYRYSVY	HLA-DPA1*01:03/DPB1*04:01	3.4			
12-26LHELSSALEIPYDEL	HLA-DQA1*01:01/DQB1*02:01	3.6			
80-94RYSVYGTTLEKLTNK	HLA-DPA1*01:03/DPB1*04:01	3.8			
67-81LRFYSKVSEFRWYRY	HLA-DPA1*01:03/DPB1*04:01	4			
42-56VLDFAFTDLTIVYRD	HLA-DQA1*01:01/DQB1*05:01	4.2			
71-85SKVSEFRWYRYSVYG	HLA-DPA1*01:03/DPB1*04:01	4.4			
34-48KGQLTETEVLDFAFT	HLA-DQA1*01:01/DQB1*02:01	4.7			
10-24RKLHELSSALEIPYD	HLA-DQA1*01:01/DQB1*02:01	4.9			
11-25KLHELSSALEIPYDE	HLA-DQA1*01:01/DQB1*02:01	5			

Table S7. The HLA-I predicted epitopes of HPV-33 E6

HPV-33 E6 Reference			HPV-33 E6 Variant		
epitopes	allele	score	epitopes	allele	score
45-54FAFADLTVVY	HLA-B*46:01	0.01	86-94HTLEQTVNK	HLA-A*11:01	0.01
45-54FAFADLTVVY	HLA-C*03:02	0.03	86-94HTLEQTVNK	HLA-A*11:01	0.01
85-94GNTLEQTVKK	HLA-A*11:01	0.03	85-94GHTLEQTVNK	HLA-A*11:01	0.02
45-54FAFADLTVVY	HLA-B*15:02	0.04	85-94GNTLEQTVNK	HLA-A*11:01	0.02
47-54FADLTVVY	HLA-C*03:02	0.05	81-88YSVYGHTL	HLA-C*03:04	0.03
86-94NTLEQTVKK	HLA-A*11:01	0.06	80-88NYSVYGHTL	HLA-A*24:02	0.04
46-54AFADLTVVY	HLA-B*15:02	0.07	86-94NTLEQTVNK	HLA-A*11:01	0.05
69-76FLSKISEY	HLA-B*15:02	0.07	69-76FLSKITEY	HLA-B*15:02	0.07
81-88YSVYGNTL	HLA-C*03:04	0.07	69-77FLSKITEYR	HLA-A*33:03	0.11
45-53FAFADLTVV	HLA-C*03:04	0.08	82-94SVYGHTLEQTVNK	HLA-A*11:01	0.13
82-93SVYGNTLEQTVK	HLA-A*11:01	0.09	88-96LEQTVNKPL	HLA-B*40:01	0.13
52-60VVYREGNPF	HLA-B*46:01	0.09	82-94SVYGHTLEQTVNK	HLA-A*11:01	0.13
52-60VVYREGNPF	HLA-B*15:02	0.12	35-43NPLQRSEVY	HLA-B*15:02	0.14
47-54FADLTVVY	HLA-B*46:01	0.12	81-88YSVYGHTL	HLA-C*08:01	0.14
82-94SVYGNTLEQTVKK	HLA-A*11:01	0.13	82-94SVYGNTLEQTVNK	HLA-A*11:01	0.15
45-53FAFADLTVV	HLA-C*08:01	0.13	69-76FLSKITEY	HLA-B*46:01	0.16
127-135NISGRWAGR	HLA-A*33:03	0.14	81-88YSVYGHTL	HLA-C*03:02	0.16
35-43KPLQRSEVY	HLA-B*15:02	0.14	79-88YNYSVYGHTL	HLA-A*24:02	0.2
80-88NYSVYGNTL	HLA-A*24:02	0.15	81-88YSVYGHTL	HLA-C*01:02	0.24
52-60VVYREGNPF	HLA-C*03:02	0.15	69-76FLSKITEY	HLA-C*03:02	0.33

Continued table 1: Table S7. The HLA-I predicted epitopes of HPV-33 E6

HPV-33 E6 Reference			HPV-33 E6 Variant		
epitopes	allele	score	epitopes	allele	score
69-77FLSKISEYR	HLA-A*33:03	0.17	84-94YGHTLEQTVNK	HLA-A*11:01	0.33
45-53FAFADLTVV	HLA-C*03:02	0.17	84-94YGNTLEQTVNK	HLA-A*11:01	0.33
46-55AFADLTVVYR	HLA-A*33:03	0.18	78-88HYNYSVYGHTL	HLA-A*24:02	0.37
47-54FADLTVVY	HLA-C*08:01	0.18	113-121REKKRHVDL	HLA-B*40:01	0.41
69-76FLSKISEY	HLA-B*46:01	0.19	68-77RFLSKITEYR	HLA-A*33:03	0.42
88-96LEQTVKKPL	HLA-B*40:01	0.2	82-92SVYGHTLEQTV	HLA-A*02:01	0.42
47-54FADLTVVY	HLA-B*15:02	0.2	68-76RFLSKITEY	HLA-B*15:02	0.45
45-53FAFADLTVV	HLA-B*46:01	0.2	68-76RFLSKITEY	HLA-A*24:02	0.49
46-54AFADLTVVY	HLA-B*46:01	0.23	82-90SVYGHTLEQ	HLA-A*11:01	0.54
19-28LETTIHNIEL	HLA-B*40:01	0.24	86-94HTLEQTVNK	HLA-A*33:03	0.58
81-88YSVYGNTL	HLA-C*08:01	0.24	86-94HTLEQTVNK	HLA-A*33:03	0.58
126-135HNISGRWAGR	HLA-A*33:03	0.25	68-76RFLSKITEY	HLA-C*03:02	0.59
18-26ALETTIHNI	HLA-A*02:01	0.25	83-94VYGHTLEQTVNK	HLA-A*11:01	0.6
11-19TLHDLCQAL	HLA-A*02:01	0.27	83-94VYGNTLEQTVNK	HLA-A*11:01	0.6
124-132RFHNISGRW	HLA-A*24:02	0.27	84-92YGHTLEQTV	HLA-C*08:01	0.62
46-54AFADLTVVY	HLA-C*03:02	0.31	91-99TVNKPLNEI	HLA-C*08:01	0.62
2-12FQDTEEKPRTL	HLA-C*08:01	0.31	91-99TVNKPLNEI	HLA-B*13:01	0.63
84-94YGNTLEQTVKK	HLA-A*11:01	0.33	68-76RFLSKITEY	HLA-B*46:01	0.71
81-88YSVYGNTL	HLA-C*03:02	0.35	87-96TLEQTVNKPL	HLA-B*40:01	0.72
47-55FADLTVVYR	HLA-A*33:03	0.37	86-94NTLEQTVNK	HLA-A*33:03	0.73
79-88YNYSVYGNTL	HLA-A*24:02	0.37	73-81ITEYRHYNY	HLA-C*03:02	0.76
21-28TTIHNIEL	HLA-C*03:04	0.39	91-102TVNKPLNEILIR	HLA-A*33:03	0.76
11-19TLHDLCQAL	HLA-C*01:02	0.39	141-149RSRRIETAL	HLA-C*03:04	0.76
42-50VYDFAFADL	HLA-A*24:02	0.4	80-88NYSVYGHTL	HLA-C*01:02	0.78
84-92YGNTLEQTV	HLA-C*08:01	0.4	86-96HTLEQTVNKPL	HLA-B*40:01	0.79
69-76FLSKISEY	HLA-C*03:02	0.41	86-96HTLEQTVNKPL	HLA-B*40:01	0.79
18-26ALETTIHNI	HLA-B*13:01	0.41	91-99TVNKPLNEI	HLA-C*03:04	0.81
2-12FQDTEEKPRTL	HLA-B*13:01	0.41	91-99TVNKPLNEI	HLA-C*03:04	0.81
81-88YSVYGNTL	HLA-C*01:02	0.41	83-92VYGHTLEQTV	HLA-A*24:02	0.82
91-102TVKKPLNEILIR	HLA-A*33:03	0.43	81-88YSVYGHTL	HLA-B*46:01	0.88
5-12TEEKPRTL	HLA-B*40:01	0.44	77-88RHYNYSVYGHTL	HLA-A*24:02	0.91
52-60VVYREGNPF	HLA-C*03:04	0.44	91-99TVNKPLNEI	HLA-C*01:02	1
5-15TEEKPRTLHDL	HLA-B*40:01	0.46			
1-10MFQDTEEKPR	HLA-A*33:03	0.48			
37-45LQRSEVYDF	HLA-B*13:01	0.48			
68-76RFLSKISEY	HLA-B*15:02	0.49			
113-121QEKKRHVDL	HLA-B*40:01	0.49			
68-77RFLSKISEYR	HLA-A*33:03	0.51			
37-45LQRSEVYDF	HLA-B*15:02	0.51			
21-28TTIHNIEL	HLA-C*08:01	0.51			

Continued table 2: Table S7. The HLA-I predicted epitopes of HPV-33 E6

HPV-33 E6 Reference			HPV-33 E6 Variant		
epitopes	allele	score	epitopes	allele	score
78-88HYNYSVYGNTL	HLA-A*24:02	0.54			
45-53FAFADLTVV	HLA-A*02:01	0.55			
73-81ISEYRHYNY	HLA-C*03:02	0.57			
83-94VYGNTLEQTVKK	HLA-A*11:01	0.58			
124-132RFHNISGRW	HLA-B*58:01	0.59			
53-60VYREGNPF	HLA-A*24:02	0.59			
44-55DFAFADLTVVYR	HLA-A*33:03	0.61			
55-65REGNPFGICKL	HLA-B*40:01	0.61			
39-47RSEVYDFAF	HLA-B*58:01	0.62			
11-19TLHDLCQAL	HLA-B*13:01	0.62			
82-92SVYGNTLEQTV	HLA-A*02:01	0.63			
11-19TLHDLCQAL	HLA-B*46:01	0.63			
53-62VYREGNPFGI	HLA-A*24:02	0.63			
11-19TLHDLCQAL	HLA-B*15:02	0.65			
68-76RFLSKISEY	HLA-A*24:02	0.65			
44-54DFAFADLTVVY	HLA-B*46:01	0.67			
11-19TLHDLCQAL	HLA-C*03:04	0.67			
68-76RFLSKISEY	HLA-C*03:02	0.68			
2-12FQDTEEKPRTL	HLA-C*03:04	0.68			
45-55FAFADLTVVYR	HLA-A*33:03	0.69			
100-108LIRCIICQR	HLA-A*33:03	0.69			
86-94NTLEQTVKK	HLA-A*33:03	0.7			
68-76RFLSKISEY	HLA-B*46:01	0.72			
46-54AFADLTVVY	HLA-A*24:02	0.72			
45-52FAFADLTV	HLA-C*03:04	0.72			
47-54FADLTVVY	HLA-C*03:04	0.73			
45-52FAFADLTV	HLA-C*08:01	0.73			
20-28ETTIHNIEL	HLA-C*08:01	0.75			
83-92VYGNTLEQTV	HLA-A*24:02	0.77			
97-105NEILIRCII	HLA-B*40:01	0.79			
2-12FQDTEEKPRTL	HLA-C*01:02	0.8			
74-83SEYRHYNYSV	HLA-B*40:01	0.81			
64-72KLCLRFLSK	HLA-A*11:01	0.86			
42-50VYDFAFADL	HLA-C*01:02	0.86			
20-28ETTIHNIEL	HLA-C*03:04	0.86			
4-12DTEEKPRTL	HLA-C*01:02	0.87			
21-28TTIHNIEL	HLA-C*01:02	0.89			
4-12DTEEKPRTL	HLA-C*08:01	0.9			
45-55FAFADLTVVYR	HLA-B*46:01	0.91			
141-149RSRRRETAL	HLA-C*03:04	0.91			

Continued table 3: Table S7. The HLA-I predicted epitopes of HPV-33 E6

HPV-33 E6 Reference			HPV-33 E6 Variant		
epitopes	allele	score	epitopes	allele	score
4-12DTEEKPRTL	HLA-C*03:04	0.92			
69-79FLSKISEYRHY	HLA-B*15:02	0.93			
76-84YRHYNYSVY	HLA-C*03:02	0.93			
35-43KPLQRSEVY	HLA-C*03:02	0.96			
11-19TLHDLCQAL	HLA-C*08:01	0.96			
45-54FAFADLTVVY	HLA-C*03:04	0.97			
37-45LQRSEVYDF	HLA-B*46:01	0.98			
2-12FQDTEEKPRTL	HLA-A*02:01	0.99			
141-149RSRRRETAL	HLA-C*01:02	0.99			

Table S8. The HLA-II predicted epitopes of HPV-33 E6

HPV-33 E6 Reference			HPV-33 E6 Variant		
epitopes	allele	score	epitopes	allele	score
40-54SEVYDFAFADLTVVY	HLA-DQA1*01:01/DQB1*02:01	0.26	63-77CKLCLRFLSKITEYR	HLA-DRB1*12:02	1.1
39-53RSEVYDFAFADLTVV	HLA-DQA1*01:01/DQB1*02:01	0.33	62-76ICKLCLRFLSKITEY	HLA-DRB1*12:02	1.1
41-55EVYDFAFADLTVVYR	HLA-DQA1*01:01/DQB1*02:01	0.38	64-78KLCLRFLSKITEYRH	HLA-DRB1*12:02	1.1
38-52QRSEVYDFAFADLTV	HLA-DQA1*01:01/DQB1*02:01	0.49	61-75GICKLCLRFLSKITE	HLA-DRB1*12:02	1.2
63-77CKLCLRFLSKISEYR	HLA-DRB1*12:02	0.71	65-79LCLRFLSKITEYRHY	HLA-DRB1*12:02	1.5
64-78KLCLRFLSKISEYRH	HLA-DRB1*12:02	0.75	60-74FGICKLCLRFLSKIT	HLA-DRB1*12:02	1.6
42-56VYDFAFADLTVVYRE	HLA-DQA1*01:01/DQB1*02:01	0.75	73-87ITEYRHYNYSVYGNT	HLA-DRB1*15:02	2.1
62-76ICKLCLRFLSKISEY	HLA-DRB1*12:02	0.84	72-86KITEYRHYNYSVYGN	HLA-DRB1*15:02	2.1
61-75GICKLCLRFLSKISE	HLA-DRB1*12:02	1.1	70-84LSKITEYRHYNYSVY	HLA-DRB1*15:02	2.1
37-51LQRSEVYDFAFADLT	HLA-DQA1*01:01/DQB1*02:01	1.1	71-85SKITEYRHYNYSVYG	HLA-DRB1*15:02	2.1
65-79LCLRFLSKISEYRHY	HLA-DRB1*12:02	1.2	74-88TEYRHYNYSVYGNTL	HLA-DRB1*15:02	2.1
60-74FGICKLCLRFLSKIS	HLA-DRB1*12:02	1.5	66-80CLRFLSKITEYRHYN	HLA-DRB1*12:02	2.4
36-50PLQRSEVYDFAFADL	HLA-DQA1*01:01/DQB1*02:01	1.7	64-78KLCLRFLSKITEYRH	HLA-DRB1*14:01	3.1
39-53RSEVYDFAFADLTVV	HLA-DQA1*01:01/DQB1*05:01	1.9	67-81LRFLSKITEYRHYNY	HLA-DRB1*12:02	3.3
75-89EYRHYNYSVYGNTLE	HLA-DRB1*15:02	2.1	63-77CKLCLRFLSKITEYR	HLA-DRB1*14:01	3.4
73-87ISEYRHYNYSVYGNT	HLA-DRB1*15:02	2.1	65-79LCLRFLSKITEYRHY	HLA-DRB1*14:01	4.1
72-86KISEYRHYNYSVYGN	HLA-DRB1*15:02	2.1	35-49NPLQRSEVYDFAFAD	HLA-DQA1*01:01 /DQB1*02:01	4.9
70-84LSKISEYRHYNYSVY	HLA-DRB1*15:02	2.1			
59-73PFGICKLCLRFLSKI	HLA-DRB1*12:02	2.1			
40-54SEVYDFAFADLTVVY	HLA-DQA1*01:01/DQB1*05:01	2.1			
74-88SEYRHYNYSVYGNTL	HLA-DRB1*15:02	2.1			
71-85SKISEYRHYNYSVYG	HLA-DRB1*15:02	2.1			
43-57YDFAFADLTVVYREG	HLA-DQA1*01:01/DQB1*02:01	2.1			
76-90YRHYNYSVYGNTLEQ	HLA-DRB1*15:02	2.1			
66-80CLRFLSKISEYRHYN	HLA-DRB1*12:02	2.3			
58-72NPFGICKLCLRFLSK	HLA-DRB1*12:02	2.4			
40-54SEVYDFAFADLTVVY	HLA-DPA1*01:03/DPB1*04:01	2.6			

Continued table 1: Table S8. The HLA-II predicted epitopes of HPV-33 E6

HPV-33 E6 Reference			HPV-33 E6 Variant		
epitopes	allele	score	epitopes	allele	score
41-55EVYDFAFADLTVVYR	HLA-DPA1*01:03/DPB1*04:01	2.7			
39-53RSEVYDFAFADLTVV	HLA-DPA1*01:03/DPB1*04:01	2.8			
41-55EVYDFAFADLTVVYR	HLA-DQA1*01:01/DQB1*05:01	2.9			
64-78KLCLRFLSKISEYRH	HLA-DRB1*14:01	2.9			
38-52QRSEVYDFAFADLTV	HLA-DQA1*01:01/DQB1*05:01	2.9			
63-77CKLCLRFLSKISEYR	HLA-DRB1*14:01	3			
67-81LRFLSKISEYRHYNY	HLA-DRB1*12:02	3.3			
42-56VYDFAFADLTVVYRE	HLA-DPA1*01:03/DPB1*04:01	3.4			
57-71GNPFGICKLCLRFLS	HLA-DRB1*12:02	3.9			
65-79LCLRFLSKISEYRHY	HLA-DRB1*14:01	4			
62-76ICKLCLRFLSKISEY	HLA-DRB1*14:01	4.3			
35-49KPLQRSEVYDFAFAD	HLA-DQA1*01:01/DQB1*02:01	4.6			
38-52QRSEVYDFAFADLTV	HLA-DPA1*01:03/DPB1*04:01	4.7			
42-56VYDFAFADLTVVYRE	HLA-DQA1*01:01/DQB1*05:01	4.7			

Table S9. The HLA-I predicted epitopes of HPV-52 E6

HPV-52 E6 Reference			HPV-52 E6 Variant		
epitopes	allele	score	epitopes	allele	score
86-94KTLEERVKK	HLA-A*11:01	0.01	86-94KTLEERVRK	HLA-A*11:01	0.01
18-26VLEESVHEI	HLA-A*02:01	0.03	86-94KTLKERVKK	HLA-A*11:01	0.04
127-135NIMGRWTGR	HLA-A*33:03	0.05	45-54FVFTDLRIVY	HLA-B*46:01	0.05
45-53FLFTDLRIV	HLA-A*02:01	0.06	17-27EVLEKSVHEIR	HLA-A*33:03	0.08
45-54FLFTDLRIVY	HLA-B*15:02	0.06	46-55VFTDLRIVYR	HLA-A*33:03	0.08
47-55FTDLRIVYR	HLA-A*33:03	0.06	127-135IIMGRWTGR	HLA-A*33:03	0.09
69-76FLSKISEY	HLA-B*15:02	0.07	45-54FVFTDLRIVY	HLA-B*15:02	0.1
52-60IVYRDNNPY	HLA-B*15:02	0.07	42-50VYKFVFTDL	HLA-A*24:02	0.16
44239FEDPATRPRTL	HLA-B*40:01	0.08	126-135HIIMGRWTGR	HLA-A*33:03	0.18
45-54FLFTDLRIVY	HLA-B*46:01	0.08	85-94GKTLEERVRK	HLA-A*11:01	0.19
46-55LFTDLRIVYR	HLA-A*33:03	0.11	45-54FVFTDLRIVY	HLA-C*03:02	0.2
52-60IVYRDNNPY	HLA-B*46:01	0.11	46-54VFTDLRIVY	HLA-B*15:02	0.21
81-88YSLYGKTL	HLA-C*03:04	0.11	97-105SEITIRCIM	HLA-B*40:01	0.21
82-92SLYGKTLEERV	HLA-A*02:01	0.12	18-26VLEKSVHEI	HLA-B*13:01	0.23
47-54FTDLRIVY	HLA-C*03:02	0.12	82-92SLYGKTLKERV	HLA-A*02:01	0.23
74-83SEYRHYQYSL	HLA-B*40:01	0.15	18-26VLEKSVHEI	HLA-A*02:01	0.24
20-28EESVHEIRL	HLA-B*40:01	0.15	45-55FVFTDLRIVYR	HLA-A*33:03	0.24
85-94GKTLEERVKK	HLA-A*11:01	0.15	81-89YSLYGKTLK	HLA-A*11:01	0.3
69-77FLSKISEYR	HLA-A*33:03	0.17	41-51EVYKFVFTDLR	HLA-A*33:03	0.34
52-60IVYRDNNPY	HLA-C*03:02	0.17	85-94GKTLKERVKK	HLA-A*11:01	0.34

Continued table 1: Table S9. The HLA-I predicted epitopes of HPV-52 E6

HPV-52 E6 Reference			HPV-52 E6 Variant		
epitopes	allele	score	epitopes	allele	score
18-26VLEESVHEI	HLA-B*13:01	0.17	82-91SLYGKTLKER	HLA-A*33:03	0.34
80-88QYSLYGKTL	HLA-A*24:02	0.18	88-96LEERVRKPL	HLA-B*40:01	0.34
69-76FLSKISEY	HLA-B*46:01	0.19	10-21RTLHELCEVLEK	HLA-A*11:01	0.4
37-45LQRREVYKF	HLA-B*13:01	0.19	46-54VFTDLRIVY	HLA-C*03:02	0.44
46-54LFTDLRIVY	HLA-B*15:02	0.21	45-53FVFTDLRIV	HLA-A*02:01	0.45
79-88YQYSLYGKTL	HLA-B*13:01	0.21	42-51VYKFVFTDLR	HLA-A*33:03	0.52
44206MFEDPATRPR	HLA-A*33:03	0.22	46-54VFTDLRIVY	HLA-B*46:01	0.52
126-135HNIMGRWTGR	HLA-A*33:03	0.24	19-28LEKSVHEIRL	HLA-B*40:01	0.53
42-50VYKFLFTDL	HLA-A*24:02	0.24	124-132RFHIIMGRW	HLA-A*24:02	0.59
18-26VLEESVHEI	HLA-C*08:01	0.24	82-93SLYGKTLKERVK	HLA-A*11:01	0.67
88-96LEERVKKPL	HLA-B*40:01	0.25	40-47REVYKFVF	HLA-B*40:01	0.69
73-81ISEYRHYQY	HLA-C*03:02	0.27	82-91SLYGKTLKER	HLA-A*11:01	0.69
75-83EYRHYQYSL	HLA-A*24:02	0.27	45-53FVFTDLRIV	HLA-C*03:04	0.7
97-105SEITIRCII	HLA-B*40:01	0.28	18-26VLEKSVHEI	HLA-C*08:01	0.71
17-27EVLEESVHEIR	HLA-A*33:03	0.31	18-26VLEKSVHEI	HLA-C*01:02	0.76
19-28LEESVHEIRL	HLA-B*40:01	0.31	125-135FHIIMGRWTGR	HLA-A*33:03	0.76
44362ATRPRTLHEL	HLA-C*01:02	0.33	45-53FVFTDLRIV	HLA-C*03:02	0.77
82-91SLYGKTLEER	HLA-A*33:03	0.35	79-89YQYSLYGKTLK	HLA-A*11:01	0.8
41-51EVYKFLFTDLR	HLA-A*33:03	0.35	120-128NANKRFHII	HLA-C*03:04	0.83
124-132RFHNIMGRW	HLA-A*24:02	0.35	132-140WTGRCSKCW	HLA-B*58:01	0.84
47-54FTDLRIVY	HLA-B*46:01	0.39	44-52KFVFTDLRI	HLA-A*24:02	0.86
18-26VLEESVHEI	HLA-C*01:02	0.4	120-131NANKRFHIIMGR	HLA-A*33:03	0.87
69-76FLSKISEY	HLA-C*03:02	0.41	120-128NANKRFHII	HLA-C*08:01	0.87
44519TLHELCEVL	HLA-A*02:01	0.43	82-94SLYGKTLEERVRK	HLA-A*11:01	0.88
78-88HYQYSLYGKTL	HLA-A*24:02	0.45	40-50REVYKFVFTDL	HLA-B*40:01	0.89
18-28VLEESVHEIRL	HLA-A*02:01	0.48	84-94YGKTLEERVRK	HLA-A*11:01	0.94
68-76RFLSKISEY	HLA-B*15:02	0.49	19-26LEKSVHEI	HLA-B*40:01	0.95
47-55FTDLRIVYR	HLA-A*11:01	0.5	46-54VFTDLRIVY	HLA-A*24:02	0.96
68-77RFLSKISEYR	HLA-A*33:03	0.51	82-93SLYGKTLEERVR	HLA-A*33:03	0.98
47-54FTDLRIVY	HLA-C*08:01	0.51	45-53FVFTDLRIV	HLA-B*46:01	1
81-88YSLYGKTL	HLA-C*03:02	0.53	80-89QYSLYGKTLK	HLA-A*11:01	1
46-54LFTDLRIVY	HLA-C*03:02	0.54			
45-54FLFTDLRIVY	HLA-C*03:02	0.54			
81-88YSLYGKTL	HLA-C*08:01	0.55			
44-52KFLFTDLRI	HLA-A*24:02	0.56			
45-55FLFTDLRIVYR	HLA-A*33:03	0.58			
46-54LFTDLRIVY	HLA-B*46:01	0.6			
47-54FTDLRIVY	HLA-B*15:02	0.61			
91-99RVKKPLSEI	HLA-B*13:01	0.61			
132-140WTGRCSECW	HLA-B*58:01	0.63			

Continued table 2: Table S9. The HLA-I predicted epitopes of HPV-52 E6

HPV-52 E6 Reference			HPV-52 E6 Variant		
epitopes	allele	score	epitopes	allele	score
82-93SLYGKTLEERVK	HLA-A*11:01	0.64			
68-76RFLSKISEY	HLA-A*24:02	0.65			
37-45LQRREVYKF	HLA-B*15:02	0.67			
79-88YQYSLYGKTL	HLA-A*24:02	0.67			
68-76RFLSKISEY	HLA-C*03:02	0.68			
35-43KELQRREVY	HLA-B*40:01	0.68			
2-12FEDPATRPRTL	HLA-B*13:01	0.69			
124-132RFHNIMGRW	HLA-B*58:01	0.69			
74-83SEYRHYQYSL	HLA-B*13:01	0.71			
82-94SLYGKTLEERVKK	HLA-A*11:01	0.72			
11-19TLHELCEVL	HLA-B*13:01	0.72			
68-76RFLSKISEY	HLA-B*46:01	0.72			
42-51VYKFLFTDLR	HLA-A*33:03	0.75			
1-18RTLHELCEV	HLA-A*02:01	0.75			
2-12FEDPATRPRTL	HLA-C*08:01	0.75			
78-86HYQYSLYGK	HLA-A*33:03	0.77			
125-135FHNIMGRWTGR	HLA-A*33:03	0.77			
84-94YGKTLEERVKK	HLA-A*11:01	0.78			
7-15TRPRTLHEL	HLA-C*01:02	0.78			
81-88YSLYGKTL	HLA-C*01:02	0.79			
82-91SLYGKTLEER	HLA-A*11:01	0.8			
19-26LEESVHEI	HLA-B*40:01	0.8			
36-43ELQRREVY	HLA-B*15:02	0.82			
73-81ISEYRHYQY	HLA-B*58:01	0.83			
73-81ISEYRHYQY	HLA-B*46:01	0.84			
40-47REVYKFLF	HLA-B*40:01	0.87			
8-15RPRTLHEL	HLA-C*01:02	0.89			
2-15FEDPATRPRTLHEL	HLA-B*40:01	0.9			
73-81ISEYRHYQY	HLA-B*15:02	0.92			
69-79FLSKISEYRHY	HLA-B*15:02	0.93			
37-45LQRREVYKF	HLA-A*24:02	0.94			
120-128NANKRFHNI	HLA-C*08:01	0.94			
120-128NANKRFHNI	HLA-C*03:04	0.95			
36-44ELQRREVYK	HLA-A*33:03	0.97			
97-104SEITIRCI	HLA-B*40:01	0.99			

Continued table 1: Table S10. The HLA-II predicted epitopes of HPV-52 E6

HPV-52 E6 Reference			HPV-52 E6 Variant		
epitopes	allele	score	epitopes	allele	score
39-53RREVYKFLFTDLRIV	HLA-DPA1*01:03/DPB1*04:01	0.15	40-54REVYKFVFTDLRIVY	HLA-DPA1*01:03/DPB1*04:01	0.2
40-54REVYKFLFTDLRIVY	HLA-DPA1*01:03/DPB1*04:01	0.17	41-55EVYKFVFTDLRIVYR	HLA-DPA1*01:03/DPB1*04:01	0.25
41-55EVYKFLFTDLRIVYR	HLA-DPA1*01:03/DPB1*04:01	0.18	39-53RREVYKFVFTDLRIV	HLA-DPA1*01:03/DPB1*04:01	0.25
42-56VYKFLFTDLRIVYRD	HLA-DPA1*01:03/DPB1*04:01	0.28	42-56VYKFVFTDLRIVYRD	HLA-DPA1*01:03/DPB1*04:01	0.33
43-57YKFLFTDLRIVYRDN	HLA-DPA1*01:03/DPB1*04:01	0.48	43-57YKFVFTDLRIVYRDN	HLA-DPA1*01:03/DPB1*04:01	0.61
38-52QRREVYKFLFTDLRI	HLA-DPA1*01:03/DPB1*04:01	0.52	38-52QRREVYKFVFTDLRI	HLA-DPA1*01:03/DPB1*04:01	0.72
63-77CIMCLRFLSKISEYR	HLA-DRB1*12:02	0.68	63-77CIMCLRFLSKISEYK	HLA-DRB1*12:02	0.77
64-78IMCLRFLSKISEYRH	HLA-DRB1*12:02	0.77	64-78IMCLRFLSKISEYKH	HLA-DRB1*12:02	0.83
62-76VCIMCLRFLSKISEY	HLA-DRB1*12:02	0.9	65-79MCLRFLSKISEYKHY	HLA-DRB1*12:02	1.5
37-51LQRREVYKFLFTDLR	HLA-DPA1*01:03/DPB1*04:01	0.97	37-51LQRREVYKFVFTDLR	HLA-DPA1*01:03/DPB1*04:01	1.7
61-75GVCIMCLRFLSKISE	HLA-DRB1*12:02	1.2	44-58KFVFTDLRIVYRDNN	HLA-DPA1*01:03/DPB1*04:01	2.1
65-79MCLRFLSKISEYRHY	HLA-DRB1*12:02	1.3	75-89EYRHYQYSLYGKTLK	HLA-DRB1*15:02	2.1
36-50ELQRREVYKFLFTDL	HLA-DPA1*01:03/DPB1*04:01	1.4	76-90YRHYQYSLYGKTLKE	HLA-DRB1*15:02	2.1
44-58KFLFTDLRIVYRDNN	HLA-DPA1*01:03/DPB1*04:01	1.5	121-135ANKRFHIIMGRWTGR	HLA-DRB1*12:02	2.3
60-74YGVCIMCLRFLSKIS	HLA-DRB1*12:02	1.6	123-137KRFHIIMGRWTGRCS	HLA-DRB1*12:02	2.4
41-55EVYKFLFTDLRIVYR	HLA-DRB1*14:01	1.9	122-136NKRFHIIMGRWTGRC	HLA-DRB1*12:02	2.5
75-89EYRHYQYSLYGKTLE	HLA-DRB1*15:02	2.1	66-80CLRFLSKISEYKHYQ	HLA-DRB1*12:02	2.5
73-87ISEYRHYQYSLYGKT	HLA-DRB1*15:02	2.1	36-50ELQRREVYKFVFTDL	HLA-DPA1*01:03/DPB1*04:01	2.6
72-86KISEYRHYQYSLYGK	HLA-DRB1*15:02	2.1	41-55EVYKFVFTDLRIVYR	HLA-DRB1*14:01	2.7
70-84LSKISEYRHYQYSLY	HLA-DRB1*15:02	2.1	74-88SEYKHYQYSLYGKTL	HLA-DPA1*01:03/DPB1*04:01	2.7
74-88SEYRHYQYSLYGKTL	HLA-DRB1*15:02	2.1	75-89EYKHYQYSLYGKTLE	HLA-DPA1*01:03/DPB1*04:01	2.8
71-85SKISEYRHYQYSLYG	HLA-DRB1*15:02	2.1	75-89EYRHYQYSLYGKTLK	HLA-DPA1*01:03/DPB1*04:01	2.9
76-90YRHYQYSLYGKTLEE	HLA-DRB1*15:02	2.1	42-56VYKFVFTDLRIVYRD	HLA-DRB1*14:01	3.1
66-80CLRFLSKISEYRHYQ	HLA-DRB1*12:02	2.2	76-90YKHYQYSLYGKTLEE	HLA-DPA1*01:03/DPB1*04:01	3.3
42-56VYKFLFTDLRIVYRD	HLA-DRB1*14:01	2.3	76-90YRHYQYSLYGKTLKE	HLA-DPA1*01:03/DPB1*04:01	3.5
59-73PYGVCIMCLRFLSKI	HLA-DRB1*12:02	2.8	120-134NANKRFHIIMGRWTG	HLA-DRB1*12:02	3.6
40-54REVYKFLFTDLRIVY	HLA-DQA1*01:01/DQB1*05:01	2.8	67-81LRFLSKISEYKHYQY	HLA-DRB1*12:02	3.8
74-88SEYRHYQYSLYGKTL	HLA-DPA1*01:03/DPB1*04:01	2.9	63-77CIMCLRFLSKISEYK	HLA-DRB1*14:01	4
75-89EYRHYQYSLYGKTLE	HLA-DPA1*01:03/DPB1*04:01	3	64-78IMCLRFLSKISEYKH	HLA-DRB1*14:01	4.1
64-78IMCLRFLSKISEYRH	HLA-DRB1*14:01	3.1	43-57YKFVFTDLRIVYRDN	HLA-DRB1*14:01	4.2
63-77CIMCLRFLSKISEYR	HLA-DRB1*14:01	3.2	40-54REVYKFVFTDLRIVY	HLA-DRB1*14:01	4.4
67-81LRFLSKISEYRHYQY	HLA-DRB1*12:02	3.2	119-133VNANKRFHIIMGRWT	HLA-DRB1*12:02	4.4
40-54REVYKFLFTDLRIVY	HLA-DRB1*14:01	3.2	124-138RFHIIMGRWTGRCSE	HLA-DRB1*12:02	4.7
43-57YKFLFTDLRIVYRDN	HLA-DRB1*14:01	3.3	41-55EVYKFVFTDLRIVYR	HLA-DRB1*12:02	4.8
41-55EVYKFLFTDLRIVYR	HLA-DRB1*12:02	3.4			
76-90YRHYQYSLYGKTLEE	HLA-DPA1*01:03/DPB1*04:01	3.5			
39-53RREVYKFLFTDLRIV	HLA-DQA1*01:01/DQB1*05:01	3.6			
65-79MCLRFLSKISEYRHY	HLA-DRB1*14:01	3.7			
41-55EVYKFLFTDLRIVYR	HLA-DQA1*01:01/DQB1*05:01	3.8			

Continued table 2: Table S10. The HLA-II predicted epitopes of HPV-52 E6

HPV-52 E6 Reference			HPV-52 E6 Variant		
epitopes	allele	score	epitopes	allele	score
42-56VYKFLFTDLRIVYRD	HLA-DQA1*01:01/DQB1*05:01	3.8			
42-56VYKFLFTDLRIVYRD	HLA-DRB1*12:02	3.8			
58-72NPYGVCIMCLRFLSK	HLA-DRB1*12:02	3.9			
62-76VCIMCLRFLSKISEY	HLA-DRB1*14:01	4.2			
40-54REVYKFLFTDLRIVY	HLA-DQA1*01:01/DQB1*02:01	4.3			
41-55EVYKFLFTDLRIVYR	HLA-DQA1*01:01/DQB1*02:01	4.5			
40-54REVYKFLFTDLRIVY	HLA-DRB1*12:02	4.6			
44-58KFLFTDLRIVYRDNN	HLA-DRB1*14:01	4.7			
9-23PRTLHELCEVLEESV	HLA-DQA1*01:01/DQB1*02:01	4.7			
43-57YKFLFTDLRIVYRDN	HLA-DRB1*12:02	4.7			
10-24RTLHELCEVLEESVH	HLA-DQA1*01:01/DQB1*02:01	5			

Table S11. The HLA-I predicted epitopes of HPV-58 E6

HPV-58 E6 Reference			HPV-58 E6 Variant		
epitopes	allele	score	epitopes	allele	score
84-92YGDTLEQTL	HLA-C*08:01	0.02	85-94GETLEQTLNK	HLA-A*11:01	0.02
85-94GDTLEQTLKK	HLA-A*11:01	0.03	85-94GDTLEQTLNK	HLA-A*11:01	0.02
45-54FVFADLRIVY	HLA-B*46:01	0.04	82-92SLYGETLEQTL	HLA-A*02:01	0.09
47-54FADLRIVY	HLA-C*03:02	0.05	84-92YGETLEQTL	HLA-C*08:01	0.11
46-55VFADLRIVYR	HLA-A*33:03	0.05	80-88NYSLYGETL	HLA-A*24:02	0.14
45-54FVFADLRIVY	HLA-B*15:02	0.07	86-94ETLEQTLNK	HLA-A*11:01	0.14
52-60IVYRDGNPF	HLA-B*46:01	0.08	86-94ETLEQTLNK	HLA-A*11:01	0.14
82-92SLYGDTLEQTL	HLA-A*02:01	0.09	85-92GETLEQTL	HLA-B*40:01	0.17
68-76RLLSKISEY	HLA-B*15:02	0.09	81-88YSLYGETL	HLA-C*03:04	0.22
41-51EVYDFVFADLR	HLA-A*33:03	0.11	83-92LYGETLEQTL	HLA-A*24:02	0.24
127-135NISGRWTGR	HLA-A*33:03	0.11	81-88YSLYGETL	HLA-C*08:01	0.27
46-54VFADLRIVY	HLA-B*15:02	0.11	84-94YGETLEQTLNK	HLA-A*11:01	0.28
52-60IVYRDGNPF	HLA-B*15:02	0.12	84-94YGDTLEQTLNK	HLA-A*11:01	0.28
52-60IVYRDGNPF	HLA-C*03:02	0.12	88-96LEQTLNKCL	HLA-B*40:01	0.3
68-76RLLSKISEY	HLA-B*46:01	0.12	82-94SLYGETLEQTLNK	HLA-A*11:01	0.35
74-83SEYRHYNYSL	HLA-B*40:01	0.14	82-94SLYGETLEQTLNK	HLA-A*11:01	0.35
47-55FADLRIVYR	HLA-A*33:03	0.16	82-94SLYGDTLEQTLNK	HLA-A*11:01	0.35
35-43KTLQRSEVY	HLA-B*58:01	0.17	79-88YNYSLYGETL	HLA-A*24:02	0.4
19-28LETSVHEIEL	HLA-B*40:01	0.19	86-94DTLEQTLNK	HLA-A*11:01	0.43
45-54FVFADLRIVY	HLA-C*03:02	0.19	82-92SLYGETLEQTL	HLA-B*13:01	0.48
35-43KTLQRSEVY	HLA-C*03:02	0.19	84-92YGETLEQTL	HLA-C*03:04	0.53
47-54FADLRIVY	HLA-B*46:01	0.2	82-91SLYGETLEQT	HLA-A*02:01	0.54
83-92LYGDTLEQTL	HLA-A*24:02	0.21	78-88HYNYSLYGETL	HLA-A*24:02	0.59
2-12FQDAEEKPRTL	HLA-C*03:04	0.21	91-99TLNKCLNEI	HLA-A*02:01	0.62
47-54FADLRIVY	HLA-C*08:01	0.21	81-88YSLYGETL	HLA-C*03:02	0.77

Continued table 1: Table S11. The HLA-I predicted epitopes of HPV-58 E6

HPV-58 E6 Reference			HPV-58 E6 Variant		
epitopes	allele	score	epitopes	allele	score
126-135HNISGRWTGR	HLA-A*33:03	0.22	84-92YGETLEQTL	HLA-C*01:02	0.83
18-26ALETSVHEI	HLA-A*02:01	0.23	81-88YSLYGETL	HLA-C*01:02	0.83
84-92YGDTLEQTL	HLA-C*03:04	0.24	85-96GETLEQTLNKCL	HLA-B*40:01	0.85
5-12AEEKPRTL	HLA-B*40:01	0.25	85-96GETLEQTLNKCL	HLA-B*40:01	0.85
45-55FVFADLRIVYR	HLA-A*33:03	0.25	137-145AVCWRPRRK	HLA-A*11:01	0.85
69-76LLSKISEY	HLA-B*15:02	0.25	86-94ETLEQTLNK	HLA-A*33:03	0.98
84-94YGDTLEQTLKK	HLA-A*11:01	0.26	86-94ETLEQTLNK	HLA-A*33:03	0.98
2-12FQDAEEKPRTL	HLA-C*08:01	0.26			
11-19TLHDLCQAL	HLA-A*02:01	0.27			
46-54VFADLRIVY	HLA-C*03:02	0.27			
124-132RFHNISGRW	HLA-A*24:02	0.27			
46-54VFADLRIVY	HLA-B*46:01	0.27			
84-92YGDTLEQTL	HLA-C*01:02	0.27			
5-15AEEKPRTLHDL	HLA-B*40:01	0.28			
64-72KVCLRLLSK	HLA-A*11:01	0.29			
18-26ALETSVHEI	HLA-B*13:01	0.29			
82-94SLYGDTLEQTLKK	HLA-A*11:01	0.3			
69-77LLSKISEYR	HLA-A*33:03	0.32			
80-88NYSLYGDTL	HLA-A*24:02	0.32			
75-83EYRHYNYSL	HLA-A*24:02	0.32			
42-50VYDFVFADL	HLA-A*24:02	0.32			
4-12DAEEKPRTL	HLA-C*03:04	0.32			
4-12DAEEKPRTL	HLA-C*08:01	0.32			
35-43KTLQRSEVY	HLA-B*46:01	0.33			
47-54FADLRIVY	HLA-B*15:02	0.35			
45-53FVFADLRIV	HLA-A*02:01	0.35			
52-60IVYRDGNPF	HLA-C*03:04	0.36			
81-88YSLYGDTL	HLA-C*08:01	0.37			
21-29TSVHEIELK	HLA-A*11:01	0.39			
2-12FQDAEEKPRTL	HLA-B*13:01	0.39			
11-19TLHDLCQAL	HLA-C*01:02	0.39			
81-88YSLYGDTL	HLA-C*03:04	0.4			
82-93SLYGDTLEQTLK	HLA-A*11:01	0.42			
39-47RSEVYDFVF	HLA-B*58:01	0.42			
68-76RLLSKISEY	HLA-C*03:02	0.42			
86-94DTLEQTLKK	HLA-A*11:01	0.43			
54-62YRDGNPFAV	HLA-C*08:01	0.44			
88-96LEQTLKKCL	HLA-B*40:01	0.46			
82-92SLYGDTLEQTL	HLA-B*13:01	0.46			
36-43TLQRSEVY	HLA-B*15:02	0.47			

Continued table 2: Table S11. The HLA-I predicted epitopes of HPV-58 E6

HPV-58 E6 Reference			HPV-58 E6 Variant		
epitopes	allele	score	epitopes	allele	score
37-45LQRSEVYDF	HLA-B*13:01	0.48			
113-121QEKKRHVDL	HLA-B*40:01	0.49			
21-28TSVHEIEL	HLA-C*08:01	0.49			
37-45LQRSEVYDF	HLA-B*15:02	0.51			
82-91SLYGDTLEQT	HLA-A*02:01	0.52			
68-76RLLSKISEY	HLA-B*13:01	0.52			
1-10MFQDAEEKPR	HLA-A*33:03	0.53			
73-81ISEYRHYNY	HLA-C*03:02	0.57			
35-43KTLQRSEVY	HLA-B*15:02	0.58			
35-45KTLQRSEVYDF	HLA-B*58:01	0.58			
44-55DFVFADLRIVYR	HLA-A*33:03	0.59			
124-132RFHNISGRW	HLA-B*58:01	0.59			
11-19TLHDLCQAL	HLA-B*13:01	0.62			
11-19TLHDLCQAL	HLA-B*46:01	0.63			
4-12DAEEKPRTL	HLA-C*01:02	0.64			
11-19TLHDLCQAL	HLA-B*15:02	0.65			
19-26LETSVHEI	HLA-B*40:01	0.66			
53-60VYRDGNPF	HLA-A*24:02	0.67			
11-19TLHDLCQAL	HLA-C*03:04	0.67			
100-108LIRCIICQR	HLA-A*33:03	0.69			
78-88HYNYSLYGDTL	HLA-A*24:02	0.69			
2-12FQDAEEKPRTL	HLA-C*01:02	0.69			
47-54FADLRIVY	HLA-C*03:04	0.71			
21-28TSVHEIEL	HLA-C*03:04	0.71			
137-145AVCWRPRRR	HLA-A*33:03	0.72			
132-140WTGRCAVCW	HLA-B*58:01	0.72			
45-53FVFADLRIV	HLA-C*03:04	0.73			
68-76RLLSKISEY	HLA-A*11:01	0.74			
46-54VFADLRIVY	HLA-A*24:02	0.76			
67-76LRLLSKISEY	HLA-B*15:02	0.77			
79-88YNYSLYGDTL	HLA-A*24:02	0.77			
20-28ETSVHEIEL	HLA-C*08:01	0.77			
35-43KTLQRSEVY	HLA-A*11:01	0.79			
34-43KKTLQRSEVY	HLA-B*58:01	0.79			
45-53FVFADLRIV	HLA-C*03:02	0.79			
97-105NEILIRCII	HLA-B*40:01	0.79			
40-50SEVYDFVFADL	HLA-B*40:01	0.84			
68-77RLLSKISEYR	HLA-A*33:03	0.88			
60-68FAVCKVCLR	HLA-A*33:03	0.88			
69-76LLSKISEY	HLA-B*46:01	0.88			

Continued table 3: Table S11. The HLA-I predicted epitopes of HPV-58 E6

HPV-58 E6 Reference			HPV-58 E6 Variant		
epitopes	allele	score	epitopes	allele	score
84-92YGDTLEQTL	HLA-C*03:02	0.91			
40-47SEVYDFVF	HLA-B*40:01	0.95			
11-19TLHDLCQAL	HLA-C*08:01	0.96			
45-53FVFADLRIV	HLA-B*46:01	0.98			
37-45LQRSEVYDF	HLA-B*46:01	0.98			
20-28ETSVHEIEL	HLA-C*03:04	0.98			
74-83SEYRHYNYSL	HLA-B*13:01	0.99			
2-12FQDAEEKPRTL	HLA-A*02:01	0.99			

Table S12. The HLA-II predicted epitopes of HPV-58 E6

HPV-58 E6 Reference			HPV-58 E6 Variant		
epitopes	allele	score	epitopes	allele	score
39-53RSEVYDFVFADLRIV	HLA-DQA1*01:01/DQB1*02:01	0.49	75-89EYRHYNYSLYGETLE	HLA-DRB1*15:02	2.1
40-54SEVYDFVFADLRIVY	HLA-DQA1*01:01/DQB1*02:01	0.51	73-87ISEYRHYNYSLYGET	HLA-DRB1*15:02	2.1
38-52QRSEVYDFVFADLRI	HLA-DQA1*01:01/DQB1*02:01	0.65	72-86KISEYRHYNYSLYGE	HLA-DRB1*15:02	2.1
63-77CKVCLRLLSKISEYR	HLA-DRB1*12:02	0.68	74-88SEYRHYNYSLYGETL	HLA-DRB1*15:02	2.1
64-78KVCLRLLSKISEYRH	HLA-DRB1*12:02	0.71	76-90YRHYNYSLYGETLEQ	HLA-DRB1*15:02	2.1
62-76VCKVCLRLLSKISEY	HLA-DRB1*12:02	0.93			
40-54SEVYDFVFADLRIVY	HLA-DPA1*01:03/DPB1*04:01	0.94			
41-55EVYDFVFADLRIVYR	HLA-DPA1*01:03/DPB1*04:01	0.95			
39-53RSEVYDFVFADLRIV	HLA-DPA1*01:03/DPB1*04:01	0.99			
41-55EVYDFVFADLRIVYR	HLA-DQA1*01:01/DQB1*02:01	1.1			
37-51LQRSEVYDFVFADLR	HLA-DQA1*01:01/DQB1*02:01	1.1			
36-50TLQRSEVYDFVFADL	HLA-DQA1*01:01/DQB1*02:01	1.1			
65-79VCLRLLSKISEYRHY	HLA-DRB1*12:02	1.3			
61-75AVCKVCLRLLSKISE	HLA-DRB1*12:02	1.5			
42-56VYDFVFADLRIVYRD	HLA-DPA1*01:03/DPB1*04:01	1.5			
60-74FAVCKVCLRLLSKIS	HLA-DRB1*12:02	1.9			
75-89EYRHYNYSLYGDTLE	HLA-DRB1*15:02	2.1			
73-87ISEYRHYNYSLYGDT	HLA-DRB1*15:02	2.1			
72-86KISEYRHYNYSLYGD	HLA-DRB1*15:02	2.1			
70-84LSKISEYRHYNYSLY	HLA-DRB1*15:02	2.1			
74-88SEYRHYNYSLYGDTL	HLA-DRB1*15:02	2.1			
71-85SKISEYRHYNYSLYG	HLA-DRB1*15:02	2.1			
76-90YRHYNYSLYGDTLEQ	HLA-DRB1*15:02	2.1			
38-52QRSEVYDFVFADLRI	HLA-DPA1*01:03/DPB1*04:01	2.2			
35-49KTLQRSEVYDFVFAD	HLA-DQA1*01:01/DQB1*02:01	2.4			
66-80CLRLLSKISEYRHYN	HLA-DRB1*12:02	2.5			
43-57YDFVFADLRIVYRDG	HLA-DPA1*01:03/DPB1*04:01	2.5			
40-54SEVYDFVFADLRIVY	HLA-DQA1*01:01/DQB1*05:01	2.8			

Continued table 1: Table S12. The HLA-II predicted epitopes of HPV-58 E6

HPV-58 E6 Reference			HPV-58 E6 Variant		
epitopes	allele	score	epitopes	allele	score
39-53RSEVYDFVFADLRIV	HLA-DQA1*01:01/DQB1*05:01	3			
42-56VYDFVFADLRIVYRD	HLA-DQA1*01:01/DQB1*02:01	3.1			
37-51LQRSEVYDFVFADLR	HLA-DPA1*01:03/DPB1*04:01	3.2			
63-77CKVCLRLLSKISEYR	HLA-DRB1*14:01	3.4			
64-78KVCLRLLSKISEYRH	HLA-DRB1*14:01	3.5			
59-73PFAVCKVCLRLLSKI	HLA-DRB1*12:02	3.5			
41-55EVYDFVFADLRIVYR	HLA-DRB1*14:01	3.7			
36-50TLQRSEVYDFVFADL	HLA-DPA1*01:03/DPB1*04:01	3.9			
42-56VYDFVFADLRIVYRD	HLA-DRB1*14:01	4.3			
62-76VCKVCLRLLSKISEY	HLA-DRB1*14:01	4.6			
41-55EVYDFVFADLRIVYR	HLA-DQA1*01:01/DQB1*05:01	4.7			
58-72NPFAVCKVCLRLLSK	HLA-DRB1*12:02	4.7			
67-81LRLLSKISEYRHYNY	HLA-DRB1*12:02	4.8			
38-52QRSEVYDFVFADLRI	HLA-DQA1*01:01/DQB1*05:01	4.8			
42-56VYDFVFADLRIVYRD	HLA-DQA1*01:01/DQB1*05:01	4.9			
65-79VCLRLLSKISEYRHY	HLA-DRB1*14:01	5			
